# Nebulised surfactant for the treatment of severe COVID-19 in adults (COV-Surf): A structured summary of a study protocol for a randomized controlled trial

**DOI:** 10.1186/s13063-020-04944-5

**Published:** 2020-12-10

**Authors:** Ahilanandan Dushianthan, Howard Clark, Jens Madsen, Robin Mogg, Lewis Matthews, Lee Berry, Jorge Bernardino de la Serna, James Batchelor, David Brealey, Tracy Hussell, Joanna Porter, Ratko Djukanovic, Martin Feelisch, Anthony Postle, Michael P. W. Grocott

**Affiliations:** 1 Respiratory and Critical Care Theme, Southampton NIHR Biomedical Research Centre, University Hospital Southampton NHS Foundation Trust, Tremona Road, Southampton, SO16 6YD UK; 2 Faculty of Medicine, University of Southampton, University Hospital Southampton NHS Foundation Trust, Tremona Road, Southampton, SO16 6YD UK; 3 UCLH Biomedical Research Centre Infection and Immunity Theme, 149 Tottenham Court Road, London, W1T 7DN UK; 4 EGA Institute for Women’s Health, Faculty of Population Health Sciences, University College London Hospital, Room 343, Medical School Building, 74, Huntley Street, London, WC1E 6AU UK; 5 Bill and Melinda Gates Medical Research Institute, 245 Main Street, Cambridge, MA02142 USA; 6 National Heart and Lung Institute, Imperial College London, Sir Alexander Fleming Building, London, SW7 2AZ UK; 7 NIHR Imperial Biomedical Research Centre, London, SW7 2AZ UK; 8 Clinical Informatics Research Unit (CIRU), University of Southampton, MP852, University Hospital Southampton NHS Foundation Trust, Southampton, SO16 6YD UK; 9 Critical Care, University College Hospitals London, 235, Euston Road, London, NW1 2BU UK; 10 The Lydia Becker Institute for Immunology and Inflammation, The University of Manchester, Manchester, UK; 11 UCL Respiratory, University College London and Interstitial Lung Disease Service, University College London NHS Foundation Trust, London, UK

**Keywords:** COVID-19, Randomised controlled trial, protocol, surfactant, Intensive Care, mechanical ventilation, nebulisation

## Abstract

**Objectives:**

SARS-Cov-2 virus preferentially binds to the Angiotensin Converting Enzyme 2 (ACE2) on alveolar epithelial type II cells, initiating an inflammatory response and tissue damage which may impair surfactant synthesis contributing to alveolar collapse, worsening hypoxia and leading to respiratory failure. The objective of this study is to evaluate the feasibility, safety and efficacy of nebulised surfactant in COVID-19 adult patients requiring mechanical ventilation for respiratory failure.

**Trial design:**

This study is a dose-escalating randomized open-label clinical trial of 20 COVID-19 patients.

**Participants:**

This study is conducted in two centres: University Hospital Southampton and University College London Hospitals. Eligible participants are aged ≥18, hospitalised with COVID-19 (confirmed by PCR), who require endotracheal intubation and are enrolled within 24 hours of mechanical ventilation. For patients unable to consent, assent is obtained from a personal legal representative (PerLR) or professional legal representative (ProfLR) prior to enrolment. The following are exclusion criteria: imminent expected death within 24 hours; specific contraindications to surfactant administration (e.g. known allergy, pneumothorax, pulmonary hemorrhage); known or suspected pregnancy; stage 4 chronic kidney disease or requiring dialysis (i.e., eGFR < 30); liver failure (Child-Pugh Class C); anticipated transfer to another hospital, which is not a study site, within 72 hours; current or recent (within 1 month) participation in another study that, in the opinion of the investigator, would prevent enrollment for safety reasons; and declined consent or assent.

**Intervention and comparator:**

Intervention: The study is based on an investigational drug/device combination product. The surfactant product is Bovactant (Alveofact®), a natural animal derived (bovine) lung surfactant formulated as a lyophilized powder in 108 mg vials and reconstituted to 45 mg/mL in buffer supplied in a prefilled syringe. It is isolated by lung lavage and, by weight, is a mixture of: phospholipid (75% phosphatidylcholine, 13% phosphatidylglycerol, 3% phosphatidylethanolamine, 1% phosphatidylinositol and 1% sphingomyelin), 5% cholesterol, 1% lipid-soluble surfactant-associated proteins (SP-B and SP-C), very low levels of free fatty acid, lyso-phosphatidylcholine, water and 0.3% calcium. The Drug Delivery Device is the AeroFact-COVID™ nebulizer, an investigational device based on the Aerogen® Solo vibrating mesh nebulizer.

The timing and escalation dosing plans for the surfactant are as follows.

**Cohort 1:** Three patients will receive 10 vials (1080 mg) each of surfactant at dosing times of 0 hours, 8 hours and 24 hours. 2 controls with no placebo intervention.

**Cohort 2:** Three patients will receive 10 vials (1080 mg) of surfactant at dosing times of 0 hours and 8 hours, and 30 vials (3240 mg) at a dosing time of 24 hours. 2 controls with no placebo intervention.

**Cohort 3**: Three patients will receive 10 vials (1080 mg) of surfactant at a dosing time of 0 hours, and 30 vials (3240 mg) at dosing times of 8 hours and 24 hours. 2 controls with no placebo intervention.

**Cohort 4:** Three patients will receive 30 (3240 mg) vials each of surfactant at dosing times of 0 hours, 8 hours and 24 hours. 2 controls. 2 controls with no placebo intervention.

The trial steering committee, advised by the data monitoring committee, will review trial progression and dose escalation/maintenance/reduction after each cohort is completed (48-hour primary outcome timepoint reached) based on available feasibility, adverse event, safety and efficacy data. The trial will not be discontinued on the basis of lack of efficacy. The trial may be stopped early on the basis of safety or feasibility concerns.

Comparator: No placebo intervention.

All participants will receive usual standard of care in accordance with the local policies for mechanically ventilated patients and all other treatments will be left to the discretion of the attending physician.

**Main outcomes:**

The co-primary outcome is the improvement in oxygenation (PaO_2_/FiO_2_ ratio) and pulmonary ventilation (Ventilation Index (VI), where VI = [RR x (PIP − PEEP) × PaCO_2_]/1000) at 48 hours after study initiation. The secondary outcomes include frequency and severity of adverse events (AEs), Adverse Device Effects (ADEs), Serious Adverse Events (SAEs) and Serious Adverse Device Events (SADEs), change in pulmonary compliance, change in positive end-expiratory pressure (PEEP) requirement of ventilatory support at 24 and 48 hours after study initiation, clinical improvement defined by time to one improvement point on the ordinal scale described in the WHO master protocol (2020) recorded while hospitalised, days of mechanical ventilation, mechanical ventilator free days (VFD) at day 21, length of intensive care unit stay, number of days hospitalised and mortality at day 28. Exploratory end points will include quantification of SARS-CoV-2 viral load from tracheal aspirates using PCR, surfactant dynamics (synthesis and turnover) and function (surface tension reduction) from deep tracheal aspirate samples (DTAS), surfactant phospholipid concentrations in plasma and DTAS, inflammatory markers (cellular and cytokine) in plasma and DTAS, and blood oxidative stress markers.

**Randomisation:**

After informed assent, patients fulfilling inclusion criteria will be randomised to 3:2 for the treatment and control arms using an internet-based block randomization service (ALEA tool for clinical trials, FormsVision BV) in combination with electronic data collection. Randomisation will be done by the recruiting centre with a unique subject identifier specific to that centre.

**Blinding (masking):**

This is an open-labelled unblinded study.

**Numbers to be randomised (sample size):**

The total sample size is 20 COVID-19 mechanically ventilated patients (12 intervention; 8 control).

**Trial Status:**

Current protocol version is V2 dated 5^th^ of June 2020. The recruitment is currently ongoing and started on the 14^th^ of October 2020. The anticipated study completion date is November 2021.

**Trial registration:**

ClinicalTrials.gov: NCT04362059 (Registered 24 April 2020), EUDAMED number: CIV-GB-20-06-033328, EudraCT number: 2020-001886-35 (Registered 11 May 2020)

**Full protocol:**

The full protocol is attached as an additional file, accessible from the Trials website (Additional file 1). In the interest in expediting dissemination of this material, the familiar formatting has been eliminated; this Letter serves as a summary of the key elements of the full protocol. The study protocol has been reported in accordance with the Standard Protocol Items: Recommendations for Clinical Interventional Trials (SPIRIT) guidelines (Additional file 2).

**Supplementary Information:**

The online version contains supplementary material available at 10.1186/s13063-020-04944-5.

## Supplementary Information

**Additional file 1.** Full Study Protocol.

**Additional file 2.** SPIRIT 2013 Checklist: Recommended items to address in a clinical trial protocol and related documents.

## Data Availability

The study investigators will have access to the final trial dataset which will be available from the author upon request.

