# Supplementary material for: Nebulised surfactant for the treatment of severe COVID-19 in adults (COV-Surf): A structured summary of a study protocol for a randomized controlled trial
Source: Trials. 2020 Dec 10;21:1014. doi: 10.1186/s13063-020-04944-5 (PMC7726271; doi:10.1186/s13063-020-04944-5)
Supplement: Supplementary file 1 — Additional file 1. Full Study Protocol. [file 13063_2020_4944_MOESM1_ESM.pdf]

Full Title:

A Clinical Trial of Nebulized Surfactant for the Treatment of Severe COVID-19 in Adults (COVSurf)

Short Title:

COVID-19 Surfactant Clinical Trial / COVSurf

This protocol has regard for the HRA guidance and order of content

FULL/LONG TITLE OF THE TRIAL

A Clinical Trial of Nebulized Surfactant for the Treatment of Severe COVID-19 in Adults (COVSurf)

SHORT TRIAL TITLE / ACRONYM

COVID-19 Surfactant Clinical Trial / COVSurf

PROTOCOL VERSION NUMBER AND DATE

Version 2 05/06/2020

RESEARCH REFERENCE NUMBERS

**IRAS Number:**	282498
**EudraCT Number:**	2020-001886-35
**EUDAMED Number:**	CIV-GB-20-06-033328
**Clinical trials.gov Number:**	NCT04362059
**SPONSORS Number:**	RHM CRI0399
**FUNDERS Number:**	INV-016631

SIGNATURE PAGE

The undersigned confirm that the following protocol has been agreed and accepted and that the Chief Investigator agrees to conduct the trial in compliance with the approved protocol and will adhere to the principles outlined in the Medicines for Human Use (Clinical Trials) Regulations 2004 (SI 2004/1031), amended regulations (SI 2006/1928) and any subsequent amendments of the clinical trial regulations, GCP guidelines, the Sponsor's (and any other relevant) SOPs, and other regulatory requirements as amended. The Medical Devices Regulations 2002 (SI2002/618), The General Product Safety Regulations 2005 (SI 2005/1803), Medical Devices Directive, 20017, 93/42/EEC, ISO 14155, ISO 13485 to be followed.

I agree to ensure that the confidential information contained in this document will not be used for any other purpose other than the evaluation or conduct of the clinical investigation without the prior written consent of the Sponsor

I also confirm that I will make the findings of the trial publicly available through publication or other dissemination tools without any unnecessary delay and that an honest accurate and transparent account of the trial will be given; and that any discrepancies and serious breaches of GCP from the trial as planned in this protocol will be explained.

For and on behalf of the Trial Sponsor:

Signature: ....

Date: ....../....../......

Name (please print):

....

Position:

....

Chief Investigator:

Signature:

Date: 05/06/2020

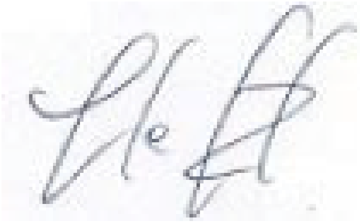

Name: (please print):

Professor Michael GROCOTT

KEY TRIAL CONTACTS

Chief Investigator	Professor Michael Grocott<br>Acute Perioperative and Critical Care Research Group<br>Southampton NIHR Biomedical Research Centre<br>CE 93, E-level, Centre Block<br>University Hospital Southampton NHS Foundation Trust<br>Tremona Road, Southampton, SO16 6YD<br>Tel: 02381 208449<br>Email: mike.grocott@soton.ac.uk
Site Study Manager - Ethics/regulatory submission and TMF	Mr Danny Pratt<br>Study Manager<br>Southampton NIHR Clinical Research Facility<br>University Hospital Southampton NHS Foundation Trust<br>Mailpoint 218<br>Tremona Road, Southampton, SO16 6YD<br>Tel: 02381 203943<br>Email: danny.pratt@uhs.nhs.uk
Project Manager (CRO)	Dr Filipa Martins<br>Head of Clinical Operations and Development<br>PHARMExcel<br>Albany Chambers, 26 Bridge Road East,<br>Welwyn Garden City, Hertfordshire AL7 1HL<br>Tel: 0203 642 6654<br>Email: filipa.martins@pharmexcel-cro.com
Sponsor Representative	Mrs Sharon Davies-Dear<br>R&D department<br>University Hospital Southampton NHS Foundation Trust<br>Tremona Road, Southampton, SO16 6YD<br>Tel: 02381 205664<br>Email: sponsor@uhs.nhs.uk
Funder(s)	The Gates Foundation<br>Farhad Imam<br>Senior Program Officer, MNCH Discovery & Tools,<br>P. O. Box 23350, Seattle, Washington 98102, USA<br>Email: Farhad.Imam@gatesfoundation.org

Key Protocol Contributors	Dr Ahilanadan Dushianthan<br>Acute Perioperative and Critical Care Research Group<br>Southampton NIHR Biomedical Research Centre<br>CE 93, E-level, Centre Block<br>University Hospital Southampton NHS Foundation Trust<br>Tremona Road, Southampton, SO16 6YD<br>Tel: 02381 208449<br>Email: a.dushianthan@soton.ac.uk<br><br>Professor (Emeritus) Anthony Postle<br>Faculty of Medicine, University of Southampton,<br>Southampton General Hospital<br>Tremona Road, Southampton, SO16 6YD<br>Tel: 02381206161<br>Email: adp@soton.ac.uk<br><br>Professor Howard Clark<br>Faculty of Population Health Sciences<br>University College London Hospital<br>Room 343, Medical School Building<br>74 Huntley Street, London, WC1E 6AU<br>Tel: 0207 679 0834<br>Email: H.Clark@ucl.ac.uk<br><br>Associate Professor Jens Madsen<br>Faculty of Population Health Sciences<br>University College London<br>Room 102, 86-96 Chenies Mews, London, WC1E 6HX<br>Tel: 0207 679 6773<br>Email: jens.madsen@ucl.ac.uk
Statistician	Robin Mogg<br>Bill and Melinda Gates Medical Research Institute<br>Address: Bill and Melinda Gates Medical Research Institute<br>245 Main Street

	Cambridge, MA 02142<br>Tel: +1-267-718-6290<br>Email: robin.mogg@gatesmri.org
Trials Pharmacist	Clinical Trials Pharmacy Department<br>University Hospital Southampton NHS Foundation Trust<br>Tremona Road, Southampton, SO16 6YD<br>Tel: 02381 206799<br>Email: clinicaltrials@uhs.nhs.uk
Chair of Data & Safety Monitoring Board	Professor Michael (Monty) Mythen<br>University College London<br>170 Tottenham Court Road, London W1T 7HA<br>Tel: 020 3447 2810<br>Email: m.mythen@ucl.ac.uk
Chair of Trial Steering Committee	Professor Stephen Brett<br>Department of Surgery & Cancer<br>Imperial College London<br>Hammersmith Hospital, London W12 0HS<br>Tel: 020 3313 4521<br>Email: stephen.brett@imperial.ac.uk
Data Management	Prof James Batchelor<br>Professorial Fellow of Clinical Informatics & Healthcare Innovation<br>Clinical Informatics Research Unit (CIRU)<br>University of Southampton, MP852, Southampton General Hospital, Southampton<br>SO16 6YD, UK<br>Email: J.Batchelor@soton.ac.uk
Study Sites	University Hospital Southampton NHS Foundation Trust<br>University College London Hospital NHS Foundation Trust

i. **LIST of CONTENTS**

GENERAL INFORMATION	Page No.
TITLE PAGE	2
RESEARCH REFERENCE NUMBERS	3
SIGNATURE PAGE	4
KEY TRIAL CONTACTS	5
i. LIST of CONTENTS	8
ii. LIST OF ABBREVIATIONS	9
iii. TRIAL SUMMARY	11
iv. FUNDING	13
v. ROLE OF SPONSOR AND FUNDER	13
vi. ROLES & RESPONSIBILITIES OF TRIAL MANAGEMENT COMMITTEES, GROUPS AND INDIVIDUALS	13
vii, PROTOCOL CONTRIBUTERS	13
viii. KEYWORDS	13
ix. TRIAL FLOW CHART	15
**SECTION**	
1. BACKGROUND	16
2. RATIONALE	16
3. OBJECTIVES AND OUTCOME MEASURES/ENDPOINTS	19
4. TRIAL DESIGN	24
5. TRIAL SETTING	26
6. PARTICIPANT ELIGIBILITY CRITERIA	26
7. TRIAL PROCEDURES	27
8. TRIAL TREATMENTS	36
9. PHARMACOVIGILANCE	41
10. STATISTICS AND DATA ANALYSIS	51
11. DATA MANAGEMENT	53
12. MONITORING, AUDIT & INSPECTION	55
13. ETHICAL AND REGULATORY CONSIDERATIONS	55
14. DISSEMINATION POLICY	59
15. REFERENCES	59
16. APPENDICIES	61

ii. LIST OF ABBREVIATIONS

Define all unusual or 'technical' terms related to the trial. Add or delete as appropriate to your trial. Maintain alphabetical order for ease of reference.

AE	Adverse Event
ADE	Adverse Device Event
APR	Annual Progress Report
AR	Adverse Reaction
BAL	Bronchoalveolar Lavage
CA	Competent Authority
CI	Chief Investigator
CRF	Case Report Form
CRO	Contract Research Organisation
CTA	Clinical Trial Authorisation
CTIMP	Clinical Trial of Investigational Medicinal Product
CTU	Clinical Trials Unit
DD	Device Deficiency
DMC	Data Monitoring Committee
DSUR	Development Safety Update Report
EC	European Commission
EMEA	European Medicines Agency
EU	European Union
EUCTD	European Clinical Trials Directive
EudraCT	European Clinical Trials Database
EUDAMED	European Database on Medical Devices
EudraVIGILANCE	European database for Pharmacovigilance
GCP	Good Clinical Practice
GMP	Good Manufacturing Practice
DSMB	Date and Safety Monitoring Board
IB	Investigator's Brochure
ICF	Informed Consent Form
ICH	The International Council for Harmonisation of Technical Requirements for Pharmaceuticals for Human
IMP	Investigational Medicinal Product
IMPD	Investigational Medicinal Product Dossier
ISF	Investigator Site File (This forms part of the TMF)

ISRCTN	International Standard Randomised Controlled Trials Number
MA	Marketing Authorisation
MHRA	Medicines and Healthcare products Regulatory Agency
MS	Member State
NHS R&D	National Health Service Research & Development
NIMP	Non-Investigational Medicinal Product
PI	Principal Investigator
PIC	Participant Identification Centre
PIS	Participant Information Sheet
QA	Quality Assurance
QC	Quality Control
QP	Qualified Person
RCT	Randomised Control Trial
REC	Research Ethics Committee
SAE	Serious Adverse Event
SADE	Serious Adverse Device Effect
SAR	Serious Adverse Reaction
SDV	Source Data Verification
SOP	Standard Operating Procedure
SmPC	Summary of Product Characteristics
SSI	Site Specific Information
SUSAR	Suspected Unexpected Serious Adverse Reaction
TMF	Trial Master File
TMG	Trial Management Group
TSC	Trial Steering Committee
USADE	Unexpected Serious Adverse Device Effect

iii.TRIAL SUMMARY

Trial Title	A Clinical Trial of Nebulised Surfactant for the treatment of severe COVID-19 in Adults.	
Internal ref. no. (or short title)	RHM CRI0399	
Clinical Phase	Pilot feasibility Phase II clinical trial	
Trial Design	Exploratory dose-escalating pilot, randomised, open-label clinical trial	
Trial Participants	Hospitalised adult patients aged ≥18 years, COVID-19 positive by PCR confirmation, and endotracheally intubated.	
Planned Sample Size	Up to 20 hospitalised adult patients (≥18 years old)	
Treatment duration	24 hours	
Follow up duration	28 days	
Planned Trial Period	June 2020 – September 2020 (4 months)	
	Objectives	Outcome Measures
Primary	1. To assess the improvement in oxygenation as determined by the PaO_2/FiO_2 ratio	Change in PaO_2/FiO_2 ratio at 48 hours after study initiation
	2. To assess the improvement in pulmonary ventilation as determined by the Ventilation Index (VI), where VI = [RR x (PIP − PEEP) × $PaCO_{2]}$/1000	Change in the Ventilation Index at 48 hours after study initiation
Secondary	1. To assess safety as judged by the frequency and severity of adverse events (AEs), Adverse Device Effects (ADEs), Serious Adverse Events (SAEs) and Serious Adverse Device Events (SADEs)	Cumulative incidence of serious adverse events (SAEs and SADEs)<br>Cumulative incidence of Grade 3 and 4 adverse events (AEs)<br>Cumulative incidence of ADEs<br>Changes in white cell count, haemoglobin, platelets, creatinine, glucose, total bilirubin, ALT, and AST over time
	2. Mean change in PaO_2/FiO_2 ratio at 24 and 48 hours after study initiation.	Change in PaO_2/FiO_2 ratio at 24 and 48 hours after study initiation

	3. Mean change in ventilatory index (VI) at 24 and 48 hours after study initiation	Change in VI at 24 hours and 48 hours after study initiation
	4. Mean change in pulmonary compliance at 24 and 48 hours after study initiation	Change in pulmonary compliance (L/cmH_2O) at 24 and 48 hours after study initiation
	5. Mean change in PEEP requirement at 24 and 48 hours after study initiation	Change in PEEP requirement at 24 and 48 hours after study initiation
	6. To evaluate clinical improvement defined by time to one improvement point on an ordinal scale, as described in the WHO master protocol (2020) daily while hospitalised and on days 15 and 28	Ordinal outcome assessed daily while hospitalised and on days 15 and 28 hospitalised<br>1. Not hospitalised, no limitations on activities<br>2. Not hospitalised, limitation on activities;<br>3. Hospitalised, not requiring supplemental oxygen;<br>4. Hospitalised, requiring supplemental oxygen;<br>5. Hospitalised, on non-invasive ventilation or high flow oxygen devices;<br>6. Hospitalised, on invasive mechanical ventilation or ECMO;<br>7. Death.
	7. Duration of mechanical ventilation	Ventilation days
	8. Ventilator free days at day 21	Number of ventilator free days at day 21
	9. Length of intensive care unit stay	Number of days of intensive care unit stay
	10. Number of days hospitalised	Hospital Days
	11. Mortality at day 28	28-day mortality (Date and cause of death (if applicable))
Investigational Medicinal Product(s)	Bovactant (Alveofact®) - bovine lung surfactant	
Formulation, Dose, Route of Administration	Lyophilized powder in 108 mg vials and reconstituted to 45 mg/mL in buffer supplied in a prefilled syringe	
Investigational Medical Device	AeroFact-COVID™ Drug Delivery System (AF-COVID)	

iv. FUNDING AND SUPPORT IN KIND

**FUNDER(S)**	**FINANCIAL AND NON FINANCIALSUPPORT GIVEN**
**Bill and Melinda Gates Foundation**	**$1,298,313.00**

v. ROLE OF TRIAL SPONSOR AND FUNDER

The Sponsor is University Hospital Southampton NHS Foundation Trust (UHS), which is the organisation that is taking legal responsibility for the trial. The Sponsor has entered into an Agreement with PHARMExcel Ltd, who will be undertaking some trial duties on behalf of the Sponsor. Roles and responsibilities for the trial are listed in an Agreement between both organisations.

The trial is funded by The Bill & Melinda Gates Foundation. The responsibilities for funding are outlined in the financial agreements between the Sponsor and the funding organisations.

vi. ROLES AND RESPONSIBILITIES OF TRIAL MANAGEMENT COMMITEES/GROUPS & INDIVIDUALS

Trial Management Committees

- Data and Safety Monitoring Board (DSMB)

The DSMB will actively monitor emerging safety and efficacy data to make recommendations about early study closure or the initiation of a larger randomised controlled clinical trial.

- Trial Management Group (TMG)

The TMG will include the Chief Investigator, clinicians and experts from relevant specialities and staff from the CRO. The TMG will be responsible for overseeing the trial and for review of all day to day study activities.

- Contract Research Organisation (CRO)

The appointed CRO will be responsible for project management of the trial (on behalf of the Sponsor). The CRO will be responsible for: the review of protocol, investigator’s brochure, associated study documents prior to initial submission and prior to any further amendments required, project management, monitoring, SAE tracking, expedited safety reporting. The CRO will liaise with the Sponsor and the Chief Investigator for any project management decisions.

- UHS - CRF Project Management Team

The assigned Study Manager will be responsible for Ethics and Regulatory submissions and for the Administration of the Trial (on behalf of the Sponsor). The Study Manager will liaise with the Sponsor, CRO Project Manager and Chief Investigator for any project submissions and administration decisions.

vii. Protocol contributors

The protocol has been developed by the Chief Investigator (Dr Michael Grocott) and principal / co-investigators.

The Protocol has been reviewed by University Hospital Southampton NHS Foundation Trust R&D department as part of the sponsorship process.

The protocol will be submitted to the UK HRA/REC and MHRA for regulatory approval.

viii. TRIAL FLOW CHART

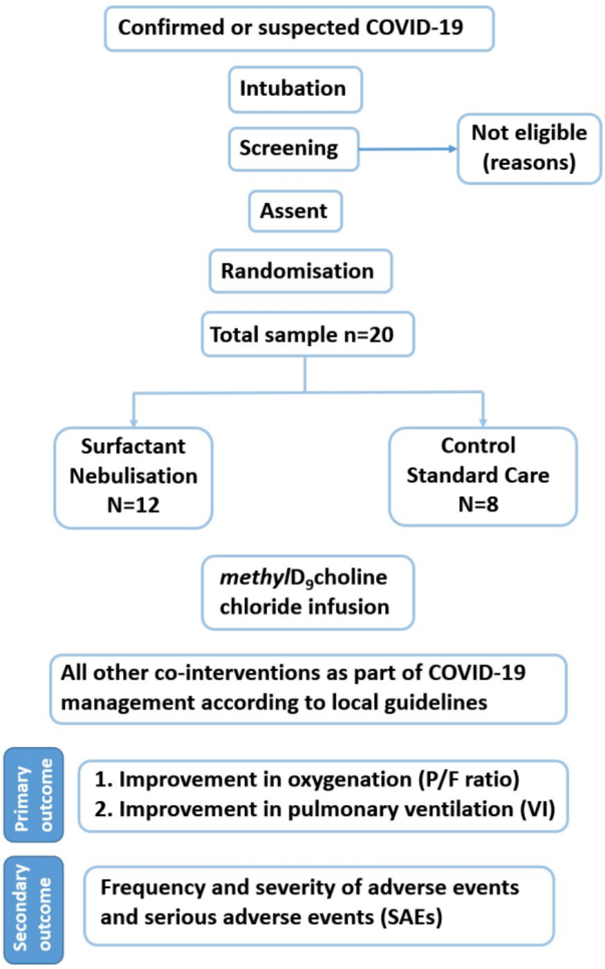

Summary of surfactant and D_9choline administration and sample collection schedule for the experimental mechanistic analyses

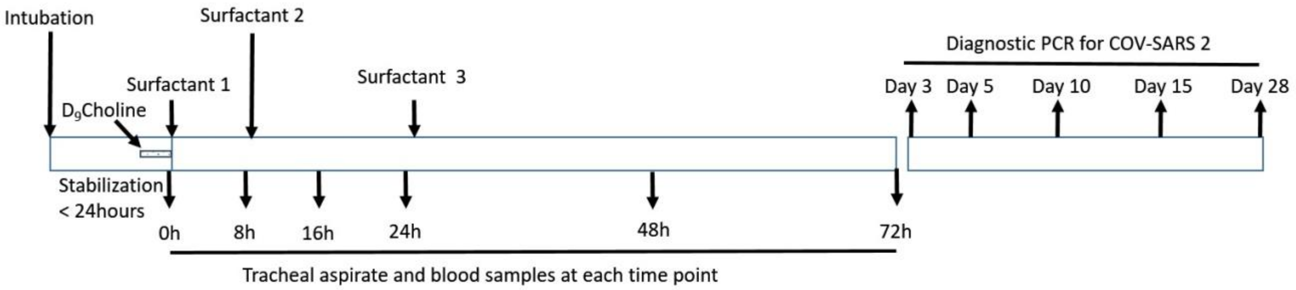

1. Surfactant will be given after a variable period of stabilization following intubation and ventilation.
2. D_9choline infusion will commence after intubation and ventilation and typically before the first surfactant nebulization – 3.0 mg/kg body weight over 3 hours.
3. Samples will be taken shortly before surfactant administration at the times indicated.
4. Lung samples – 20-50 ml saline by ET tube followed by closed deep suction tracheal aspirates. 5-10 ml recovery expected.
5. EDTA blood samples – 10 ml; Transfix blood sample – 9 ml.

1 BACKGROUND

In December 2019, the Wuhan Municipal Health Committee identified an outbreak of viral pneumonia cases of unknown cause. Coronavirus RNA was quickly identified in some of these patients. This novel coronavirus has been designated SARS-CoV-2, and the disease caused by this virus has been designated COVID-19. This disease has now spread globally with a particularly high burden of disease in Europe and the USA.

Currently, there are no approved therapeutic agents available for coronaviruses. Initial epidemiological, radiographic, and pathological data from Wuhan in combination with basic cellular and molecular biologic data suggest that early surfactant dysfunction, may be a specific aspect of the high-morbidity coronaviruses SARS-CoV-1, MERS, and SARS-CoV-2. Accordingly, the possibility that early surfactant replacement could be beneficial in preventing progression of disease severity is especially compelling given the established safety profile of surfactant, which although officially indicated for neonates, has been used in many adult Acute Respiratory Distress Syndrome (ARDS) studies.

Lung surfactant is a lipoprotein complex comprising approximately 90% lipid, of which phosphatidylcholine (PC) is the principal component, with four specific surfactant proteins (SP-A, SP-B, SP-C, SP-D). Synthesis and secretion of the functional surfactant complex is uniquely confined to the type II cell (AT-II) of the alveolar epithelium (Goss 2013). Lung surfactant forms a monolayer covering the alveolar surface where it reduces the work of breathing by opposing surface tension forces and preventing alveolar collapse. The high surface pressure caused by surfactant is a major factor in minimizing the volume of the epithelial ling fluid of the alveoli and terminal bronchioles and preventing lung oedema. SP-B and SP-B promote formation of the surfactant layer at the air:liquid interface, while SP-A and SP-D are in the first line defense against microbial infection and bind a number of viruses including influenza.

The hypothesis behind the proposed trial of surfactant therapy for COVID-19 infected patients requiring ventilator support is that endogenous surfactant is dysfunctional. This could be due to decreased concentration of surfactant phospholipid and protein, altered surfactant phospholipid composition, surfactant protein proteolysis and/or oedema protein inhibition of surfactant surface tension function and/or oxidative inactivation of surfactant proteins. Variations of these dysfunctional mechanisms have been reported in a range of lung diseases, including cystic fibrosis and severe asthma (Postle 1999), and in child and adult patients with ARDS. Our studies of surfactant metabolism in adult ARDS patients showed altered percentage composition of surfactant PC, with decreased DPPC and increased surface tension-inactive unsaturated species, and decreased concentrations of both total PC and phosphatidylglycerol (PG) (Schmidt 2007, Dushianthan 2014).

The SARS-CoV-2 virus binds to the angiotensin converting enzyme-2 (ACE2) receptor (Hoffman 2020), which is preferentially expressed in the peripheral lung ATII cells (Qi 2020). Consequent viral infection of ATII cells could reduce cell number and impair the capacity of the lungs to synthesise and secrete surfactant. This, however, has not yet been demonstrated empirically in COVID-19 patients. If this is the case, then exogenous surfactant administration to the lungs is potential one treatment option to mitigate disease severity in these patients.

2 RATIONALE

Hypothesis: A deficiency of functionally intact surfactant contributes to the deterioration in pulmonary function in patients with severe COVID-19.

Assuming that this hypothesis is correct, the dosing of surfactant and the number of administrations required to correct such a deficiency are unknown. Therefore, the feasibility component of this study

includes within-patient dose escalation based upon practical considerations, extrapolation from neonatal RDS, and prior adult ARDS studies. Dosing in the main stage of the study will be decided based on the responses observed in the feasibility stage.

The rationale for this choice of intervention is based upon:

- The use by SARS-CoV-2 of cell surface ACE2 to infect host cells (Hoffman, 2020).
- Single-cell transcriptomic data demonstrating the preferential expression of ACE2 in ATII cells, which are the source of pulmonary surfactant (Travaglini 2020).
- The clinical and radiographic features of COVID-19, which closely resemble those of neonatal respiratory distress syndrome, include ground-glass opacities and air bronchograms on chest radiography (Shi 2020) and hyaline membranes at postmortem (Xu 2020) and unpublished data (from a case in Milan).
- The clinical efficacy of pulmonary surfactant in neonatal respiratory distress syndrome (RDS) the underlying cause of which is surfactant deficiency.
- A case series indicating clinical efficacy of pulmonary surfactant in patients <14 years old with ARDS associated with underlying viral but not bacterial infection (Moller 2003).

Previous studies of surfactant therapy in adults, either natural (Walmrath 2002) or synthetic (Spragg 2004), have shown neither convincing benefit nor harm. Pooled analysis of synthetic surfactant therapy in ARDS suggests potential benefit in patients with pneumonia or aspiration (Taut 2008). However, there is reason to believe that evaluating surfactant therapy in COVID-19 might show benefit:

(i) The current proposed trial will use a modified nebulizer (investigational device) that generates particles of sufficiently small diameter with potential to deliver significantly larger surfactant volumes for effective delivery to the alveoli. Nebulizers used in previous trials by contrast were very ineffective at alveolar delivery (Weg 1994, Anzueto 1996).

(ii) The natural bovine product containing lipid, SP-B and SP-C is more dilute than alternative preparations, has a lower viscosity and repeat administrations can be delivered without interrupting the ventilator circuit.

(iii) Previous clinical trials investigated heterogeneous populations with a range of causes precipitating ARDS, including lung infection, sepsis, endotoxic shock and near drowning (Taut 2008). By contrast, COVID-19 patients represent a relatively homogeneous study population with alveolar rather than systemic injury.

(iv) The hypothesis that SARS-COV-2 may invade ATII cells, leading to reduced surfactant production contributing to the pathogenesis of COVID-19.

(v) Surfactant components with reported specific anti-viral effects, such as PG (Voelker 2019), may also be active against SARS-COV-2.

The rationale behind the proposed trial of surfactant therapy for COVID-19 infected patients requiring ventilator support is that endogenous surfactant is dysfunctional. This could be due to decreased concentration of surfactant phospholipid and protein, altered surfactant phospholipid composition, surfactant protein proteolysis and/or oedema protein inhibition of surfactant surface tension function. However, there is no empirical evidence to establish any of these mechanisms. Mechanistic approaches to address unanswered questions are set out below. These questions include:

- Is the concentration/composition of pulmonary surfactant phospholipid and protein altered in patients with COVID-19? Surfactant phosphatidylcholine (PC) is enriched in dipalmitoyl PC (DPPC) and phosphatidylglycerol (PG). ARDS patients exhibit altered percentage composition of surfactant PC, with decreased DPPC and increased surface tension-inactive unsaturated species, and decreased concentrations of both total PC and PG (Schmidt 2007, Dushianthan 2014). SP-A and SP-D concentrations are decreased in a variety of lung diseases including cystic fibrosis and severe asthma (Postle 1999), with increased SP-D concentration in plasma. Additionally, proteolytic fragments of SP-D are detected in lung infections.
- Are the surface tension-lowering properties of BAL/ tracheal suction derived surfactant impaired?
- Does viral infection decrease synthesis of surfactant phospholipid and protein by ATII cells?
- Does SAR-COV2 infection reduce ATII cell numbers in the lungs?
- Can SP-D bind SARS-COV2? If so, does this modulate the viral response?
- What are the features of the inflammatory response in COVID-19 infection?
- Does surfactant modulate the immune/inflammatory response?
- Does exogenous administration of surfactant combat the inhibitory activity of plasma proteins and the inflammatory reaction on endogenous surfactant function?
- Does the virus trigger enhance reactive oxygen species (ROS) production with the possibility of surfactant inactivation secondary to structural and functional alterations of surfactant protein-B and -C (Schwarz 1996; Rodriguez-Capote 2006).

2.1 Assessment and management of risk

2.1.1 Risks to participant safety in relation to the IMP

Clinical studies in the target population with instilled SF-RI 1 (under the marketing name Alveofact) show that its bronchoscopic application in adults with ARDS is safe, feasible and effective, resulting in a pronounced improvement in gas exchange (Walmrath 1996, Gunther 2002, Walmrath 2002, Wetshoff 2001). There have been no safety concerns associated with SF-RI 1 used for instillation as indicated by the Periodic Safety Update Report supplied by the legal manufacturer Alveofact (the marketing name of SF-RI 1).

Type B = somewhat higher than the risk of standard medical care

Alveofact (SF-RI), was initially approved in Germany as a medicinal product with the Marketing Authorization number 19273.00.00: Alveofact 45 mg/mL (Lyomark Pharma GmbH is the current authorization holder) for ETT instillation for the prevention of RDS in prematurely born infants. Alveofact is approved in 6 EU member states: Germany, Austria, Croatia, Hungary, Latvia and Lithuania and in 24 countries outside the EU.

SF RI 1 will be sourced from Lyomark Pharma GmbH and supplied to the clinical study sites as a lyophilized formulation of 108 mg, along with the required diluent for reconstitution, and labelled for clinical investigational use only.

2.1.2 Risks to participant in relation to IMP/Device combination

The AeroFact-COVID™ (AF-COVID) combination product system (nebuliser) is not a marketed product in-itself.

Regarding administration of AF-COVID to intubated, mechanically ventilated adult patients, the risks are in 3 groups:

1. Risks associated with the use of the Device are controlled in the Warning Section of the Instructions for Use. (DFU. Quick start Guide and Insreuctions for use, page 3-4).

2. Risks associated with SF-RI 1 surfactant are risks related to bolus instillation delivery via the endotracheal tube.

3. Risks associated with the combination product:
- Hypoventilation, hypoxemia
- Pneumothorax (associated with treatment related changes in lung compliance)
- Progression of COVID-19-related ARDS
- Irritation of the airways
- Introduction of pathogens other than COVID-19 to the respiratory tract
- Further dissemination of COVID-19 by aerosol

These clinical risks will be properly controlled and/or mitigated relative to the device design e.g. verification testing or labelling (instructions, warnings and cautions). Furthermore, the drug delivery system will be tested for its safety following ISO and IEC standards for materials, biological and electrical safety, and electromagnetic compatibility.

Since there is no current clinical experience with AeroFact-COVID™ combination product, there are no observed expected adverse reactions that can serve the investigators as guidance for reporting serious adverse events in the clinical study. Therefore, all treatment related Serious Adverse Events (SAEs) will be considered unexpected and will be reported to the authorities as per national requirements.

Please refer to the Investigators Brochure – Reference Safety Information Section - for a detailed list of clinical hazards identified in the initial risk assessment based on the nature of the product and the target population.

There is no guarantee that adult patients exhibiting ARDS symptoms due to lung infection with SARS CoV-2 will receive direct benefit from AeroFact-COVID™. The benefits of AF-COVID may be an improvement of lung function and decreased need for escalation of respiratory support.

Given SARS-CoV-2, the virus causing CODIV-19, is now a global pandemic claiming thousands of lives across the world, the Sponsor "University Hospital Southampton NHS Foundation Trust" and Aerogen Pharma believe that the potential benefits outweigh the potential risks with AF-COVID combination product.

3 OBJECTIVES AND OUTCOME MEASURES/ENDPOINTS

3.1 Primary objective

A co-primary endpoint of reduction in either the Oxygenation index or Ventilatory index in patients receiving surfactant therapy.

The co-primary efficacy objectives are:

1. To assess the improvement in oxygenation as determined by the PaO_2/FiO_2 ratio 48 hours after study initiation
2. To assess the improvement in pulmonary ventilation as determined by the Ventilation Index (VI), where VI = [RR x (PIP − PEEP) × $PaCO_2$]/1000 at 48 hours after study initiation

3.2 Secondary objectives

The key secondary objective is:

1. To assess safety as judged by the frequency and severity of adverse events (AEs), Adverse Device Effects (ADEs), Serious Adverse Events (SAEs) and Serious Adverse Device Events (SADEs)

Other secondary objectives are:

2. Mean change in in PaO_2/FiO_2 ratio at 24 and 48 hours after study initiation
3. Mean change in ventilatory index at 24 and 48 hours after study initiation
4. To assess the change in pulmonary compliance at 24 and 48 hours after study initiation
5. To assess the change in PEEP requirement at 24 and 48 hours after study initiation
6. To evaluate clinical improvement defined by time to one improvement point on an ordinal scale, as described in the WHO master protocol (2020) daily while hospitalised and on days 15 and 28
7. Duration of days of mechanical ventilation
8. Mechanical ventilator free days (VFD) at day 21
9. Length of intensive care unit stay
10. Number of days hospitalised
11. Mortality at day 28

3.3 Outcome measures/endpoints

3.3.1 Primary Endpoint

The co-primary endpoints are:

1. Change in PaO_2/FiO_2 ratio 48 hours after study initiation
2. Change in the Ventilation Index 48 hours after study initiation

3.3.2 Secondary Endpoints

The key secondary endpoint is safety over the first 28 days after administration of surfactant assessed by:

1. Cumulative incidence of serious adverse events (SAEs and SADEs);
   Cumulative incidence of Grade 3 and 4 adverse events (AEs)
   Cumulative incidence of ADEs and
   Changes in white cell count, hemoglobin, platelets, creatinine, glucose, total bilirubin, ALT, and AST over time.

Other secondary endpoints are:

2. Change in PaO_2/FiO_2 at 24 and 48 hours after study initiation
3. Change in value of Ventilation Index at 24 and 48 hours after study initiation
4. Change in pulmonary compliance (L/cmH_2O) at 24 and 48 hours after study initiation

5. Change in PEEP requirement at 24 and 48 hours after study initiation
6. Ordinal outcome assessed daily while hospitalised and on days 15 and 28

    1. *Not hospitalised, no limitations on activities*
    2. *Not hospitalised, limitation on activities;*
    3. *Hospitalised, not requiring supplemental oxygen;*
    4. *Hospitalised, requiring supplemental oxygen;*
    5. *Hospitalised, on non-invasive ventilation or high flow oxygen devices;*
    6. *Hospitalised, on invasive mechanical ventilation or ECMO;*
    7. *Death.*

7. Duration of mechanical ventilation- Number of days on mechanical ventilation
8. Mechanical ventilator free days (VFD) at day 21
9. Length of intensive care unit stay
10. Number of days hospitalised
11. Mortality at day 28 - Date and cause of death (if applicable)

3.3.3 Exploratory Endpoints

Additional exploratory endpoints include,

1. Diagnostic PCR for SARS-CoV-2 in TA samples on Days 3 (72 hrs), 5, 10, 15 and 28 while still hospitalised (quantitative measurements when available).
2. Closed suction deep tracheal aspirate (TA) samples before the first surfactant dose to obtain baseline surfactant phospholipid and protein concentrations, followed by subsequent TA samples at 8 hrs, 16 hrs, 24hrs, 48 hrs and 72 hrs. These analyses will determine efficiency of surfactant delivery to the peripheral lung.
3. Quantify improvement in surface tension reduction measurements from TA samples following surfactant administration.
4. Concentrations of surfactant phospholipids and proteins in longitudinal TA samples showing improvement following surfactant therapy
5. Reduction of inflammatory indices such as cellular and cytokine inflammatory markers in TA samples.
6. Evidence of earlier increased endogenous synthesis of surfactant (indicating recovery) following surfactant therapy.
7. Reduction of inflammatory indices such as cellular and cytokine inflammatory markers and of oxidative stress markers in blood samples.

3.4 Table of endpoints/outcomes

Objectives		Outcome Measures	Time point(s) of evaluation of this outcome measure (if applicable)
**Primary Objectives**			
1	To assess the improvement in oxygenation as determined by the PaO_2/FiO_2 ratio	Change in PaO_2/FiO_2 ratio at 48 hours after study initiation	48 hours after study initiation
2	To assess the improvement in pulmonary ventilation as determined by the Ventilation	Change in the Ventilation Index at 48	48 hours after study initiation

	Index (VI), where VI = [RR x (PIP − PEEP) × $PaCO_2$]/1000	hours after study initiation	
**Secondary Objectives**			
1	To assess safety as judged by the frequency and severity of adverse events (AEs), Adverse Device Effects (ADEs), Serious Adverse Events (SAEs) and Serious Adverse Device Events (SADEs).	Cumulative incidence of serious adverse events (SAEs) and serious adverse device events SADEs<br><br>Cumulative incidence of Grade 3 and 4 adverse events (AEs).<br><br>Cumulative incidence of ADEs.<br><br>Changes in white cell count, haemoglobin, platelets, creatinine, glucose, total bilirubin, ALT, and AST over time.	Recorded daily over the first 28 days after study initiation
2	Mean change in in PaO_2/FiO_2 ratio at 24 and 48 hours after study initiation.	Change in PaO_2/FiO_2 at 24 and 48 hours after study initiation	24 and 48 hours after study initiation
3	Mean change in ventilatory index at 24 and 48 hours after study initiation	Change in ventilatory index value at 24 and 48 hours after study initiation	24 and 48 hours after study initiation
4	To assess the change in pulmonary compliance at 24 and 48 hours after study initiation	Change in pulmonary compliance (L/cmH_2O) at 24 and 48 hours after study initiation	24 and 48 hours after study initiation
5	To assess the change in best PEEP requirement at 24 and 48 hours after study initiation	Change in PEEP requirement at 24 and 48 hours after study initiation	24 and 48 hours after study initiation
6	To evaluate clinical improvement defined by time to one improvement point on an ordinal scale, as described in the WHO master protocol (2020) daily while hospitalised and on days 15 and 28	Ordinal outcome assessed daily while hospitalised and on days 15 and 28 hospitalised<br><br>1. Not hospitalised, no limitations on activities<br><br>2. Not hospitalised, limitation on activities;	Assessed daily while hospitalised and on Days 15 and 28.

		3. Hospitalised, not requiring supplemental oxygen;<br>4. Hospitalised, requiring supplemental oxygen;<br>5. Hospitalised, on non-invasive ventilation or high flow oxygen devices;<br>6. Hospitalised, on invasive mechanical ventilation or ECMO;<br>7. Death.	
7	The number of days of mechanical ventilation	Ventilation days	Every day after study initiation
8	Mechanical ventilator free days at day 21	VFD at day 21	Every day after study initiation until day 21
9	Length of intensive care unit stay	ICU length of stay	Every day after study initiation
9	The number of days hospitalised	Hospital days	Every day after study initiation
10	The mortality at Day 28	28-day mortality	On day 28 after the study initiation

**Exploratory Endpoints**		**Time points**
1	Tracheal aspirate for viral PCR	Days 3, 5,10, 15 and 28 while still hospitalised
2	Determine efficiency of surfactant delivery to the peripheral lung and surfactant synthesis mechanisms	Closed suction deep tracheal aspirate (TA) samples:<br>- At time =0, before the first surfactant dose<br>- Subsequent 8 hrs, 16 hrs, 24hrs, 48 hrs and 72 hrs<br>- Time points will be same for the control group without surfactant administration
3	Quantify improvement in surface tension reduction measurements from TA samples following surfactant administration	Closed suction deep tracheal aspirate (TA) samples:<br>- At time =0, before the first surfactant dose<br>- Subsequent 8 hrs, 16 hrs, 24hrs, 48 hrs and 72 hrs<br>- Time points will be same for the control group without surfactant administration
4	Concentrations of surfactant phospholipids and proteins in longitudinal	Closed suction deep tracheal aspirate (TA) samples:<br>- At time =0, before the first surfactant dose

	specimens of plasma and TA samples showing improvement following surfactant therapy	- Subsequent 8 hrs, 16 hrs, 24hrs, 48 hrs and 72 hrs<br>- Time points will be same for the control group without surfactant administration
5	Reduction in inflammatory indices such as cellular and cytokine inflammatory markers in TA samples	Closed suction deep tracheal aspirate (TA) samples:<br>- At time =0, before the first surfactant dose<br>- Subsequent 8 hrs, 16 hrs, 24hrs, 48 hrs and 72 hrs<br>- Time points will be same for the control group without surfactant administration
6	Evidence of earlier increased endogenous synthesis of surfactant (indicating recovery) following surfactant therapy.	Closed suction deep tracheal aspirate (TA) samples:<br>- At time =0, before the first surfactant dose<br>- Subsequent 8 hrs, 16 hrs, 24hrs, 48 hrs and 72 hrs<br>- Time points will be same for the control group without surfactant administration
7	Reduction of inflammatory indices such as cellular and cytokine inflammatory markers and of oxidative stress markers in blood samples	Blood samples:<br>- At time =0, before the first surfactant dose<br>- Subsequent 8 hrs, 16 hrs, 24hrs, 48 hrs and 72 hrs<br>- Time points will be same for the control group without surfactant administration

4 TRIAL DESIGN

This study is an exploratory, dose-escalating pilot trial to test the feasibility, safety and preliminary efficacy of aerosolized lung delivery of surfactant, a medicine with a known safety profile, to improve the oxygenation and pulmonary compliance of adult patients diagnosed with moderate to severe COVID-19 associated lung injury with increasing oxygenation requirements.

The timing of escalating dosing and assessment of clinical response is based on the rapid oxygenation response of infants with surfactant deficiency (Collaborative European Multicenter Study Group 1988) and the clinical response described in adults after endobronchial administration of surfactant in ARDS (Walmrath 2002). Nevertheless, it is acknowledged that the optimal dose required for surfactant replacement therapy in adults and the rate of clinical response in patients with COVID-19 are unknown. Therefore, we propose a dose-escalating, adaptive trial design in which the feasibility of administration of aerosolized surfactant and the clinical response in mechanically ventilated COVID-19 patients are assessed. The dosing regimen may be adjusted based on accumulating data and may depend on the dose required to achieve a clinical response and the speed with which that response is seen.

Patients will enter the study and will be randomised to 3:2 for the treatment and control arms where they will receive three doses of Alveofact® via nebulizer or the control arm where treatment will follow standard of care.

Patients in the treatment arm will be assigned into cohorts sequentially as follows:

- **COHORT 1**: 3 patients will receive 10 vials (1080 mg) each of Alveofact® at dosing times of 0 hours, 8 hours and 24 hours. 2 controls.

- **COHORT 2**: 3 patients will receive 10 vials (1080 mg) of Alveofact® at dosing times of 0 hours and 8 hours, and 30 vials (3240 mg) of Alveofact® at a dosing time of 24 hours. 2 controls
- **COHORT 3**: 3 patients will receive 10 vials (1080 mg) of Alveofact® at a dosing time of 0 hours, and 30 vials (3240 mg) of Alveofact® at dosing times of 8 hours and 24 hours. 2 controls
- **COHORT 4**: 3 patients will receive 30 (3240 mg) vials each of Alveofact® at dosing times of 0 hours, 8 hours and 24 hours. 2 controls

Control arm:

CONTROL: Each cohort of 3 patients (in the treatment arm) will have 2 patients acting as controls (5 patients per cohort; total control n=8). The protocol for the control arm will be identical to the treatment arm except for surfactant nebulization.

After each cohort has completed treatment (or equivalent time for controls), the investigators will pause to review the outcomes of the treatment based on the co-primary outcomes (Pa02/Fi02 ratio and Ventilation index at 48 hours), reported adverse events and any additional safety or feasibility considerations. Additional trial outcome data may be taken into account if considered important in the opinion of the principal and chief investigators or if requested by the DSMB or TSC.

The decision to progress may be: 1. increase dose to next cohort level according to planned schedule (see below); 2. decrease dose to previous cohort level according to planned schedule (see below); 3. decrease dose to alternative level (specifically for cohort 1); 4. maintain dosage at current level. If there is a substantial and sustained improvement in the outcomes of a cohort, the subsequent cohorts may continue with the same dose as the previous cohort. If no improvement is observed, the subsequent cohort may increase the dosage. Early and substantial improvement during administration of the drug, or adverse events thought to be a safety concern, may prompt the investigators to reduce the administered dose during the conduct of the study.

Decisions relating to trial progression and dose escalation/maintenance/reduction will be made by the TSC after review of data by the DSMB. The DSMB will meet immediately prior to the TSC in open session but may meet in closed session at the request of any members of the DSMB or TSC. The TSC will meet after the DSMB and receive a verbal update from the DSMB. The TSC will be the final arbiter of trial progression and dose escalation/maintenance/reduction decisions. The majority of the TSC and all members of the DSMB will be independent of the trial investigators and trial conduct. An extraordinary meeting of the DSMB may be convened if the investigators have any safety concerns. Such a meeting would make recommendations to the TSC on trial continuation/discontinuation. The trial will not be discontinued on the basis of lack of efficacy. The trial may be stopped early on the basis of safety or feasibility concerns.

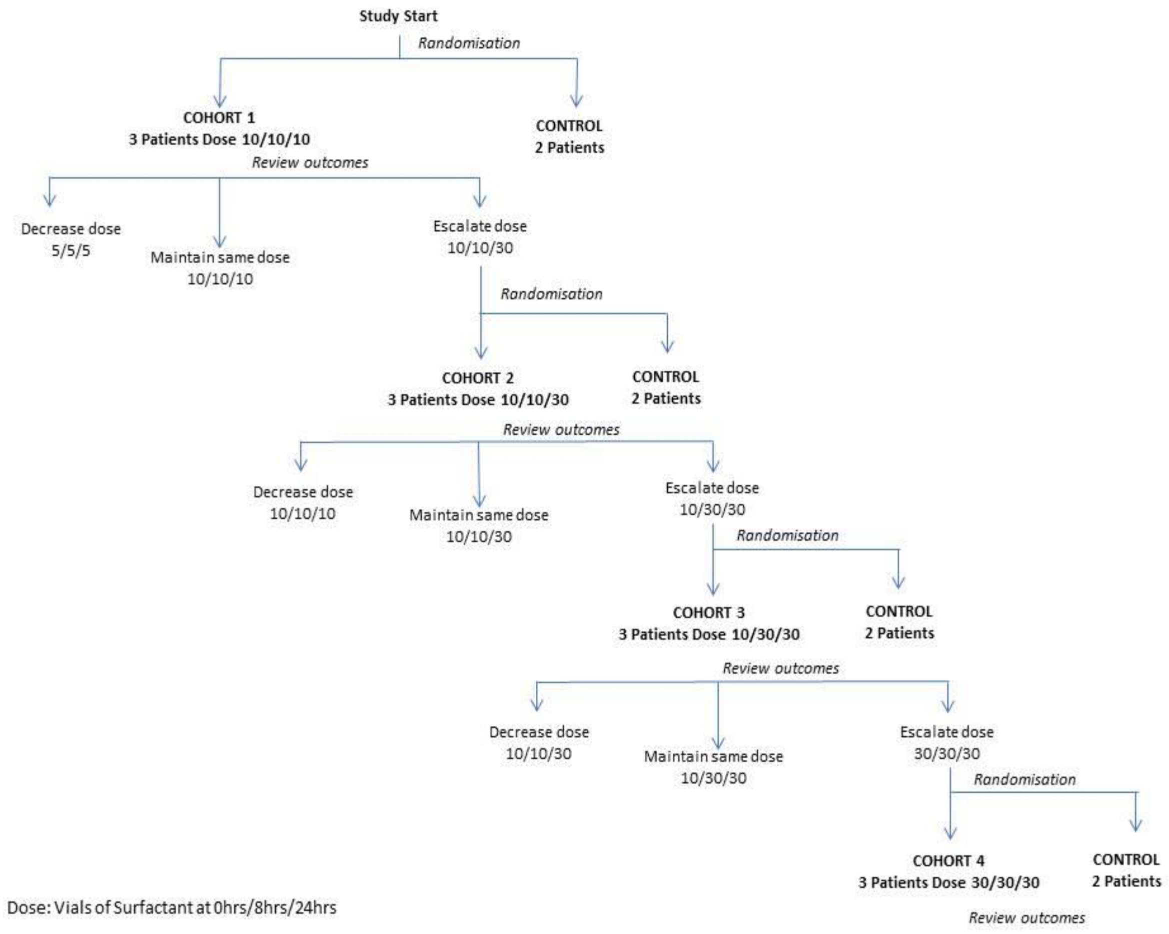

5 TRIAL SETTING

This is a multicentre, randomised, open-label clinical trial conducted at University Hospital Southampton NHS Foundation Trust and University College London Hospital NHS Foundation Trust. Adult patients diagnosed with severe COVID-19 associated lung injury with increasing oxygenation requirements will be recruited to the study from each site's inpatient population.

6 PARTICIPANT ELIGIBILITY CRITERIA

6.1 Inclusion criteria

1. Age ≥18 years old
2. Confirmed COVID-19 positive by PCR
3. Within 24 hours of mechanical ventilation
4. Assent obtained from personnel legal representative (PerLR) or professional legal representative (ProfLR)

6.2 Exclusion criteria

1. Imminent expected death within 24 hours
2. Specific contraindications to surfactant administration (e.g. known allergy, pneumothorax, pulmonary hemorrhage)

3. Known or suspected pregnancy
4. Stage 4 severe chronic kidney disease or requiring dialysis (i.e., eGFR < 30)
5. Liver failure (Child-Pugh Class C)
6. Anticipated transfer to another hospital, which is not a study site within 72 hours
7. Current participation or participation in another study within the last month that in the opinion of the investigator would prevent enrollment for safety purposes
8. Consent declined

7 TRIAL PROCEDURES

7.1 Recruitment

7.1.1 Participant identification

Hospitalised patients aged ≥18 that require endotracheal intubation will be considered for inclusion in the study.

Potential patients will be identified by an existing member of the patient's clinical care team. Participant medical records will be reviewed for consideration of inclusion / exclusion criteria. A delegated investigator will confirm inclusion / exclusion prior to enrolling a patient on the study.

7.1.2 Screening

Study participants will be hospitalised patients aged ≥18 with laboratory-confirmed SARS-CoV-2. The investigator or delegate will review the patient's medical history and medical notes for the inclusion and exclusion criteria (section 6.1 and 6.2).

Pregnancy is an exclusion criterion. Non-pregnant status will be confirmed via urine pregnancy test at screening if this has not been carried out as part of standard of care already.

7.1.3 Payment

No payment will be made to participants.

7.2 Consent

It is the responsibility of the Principal Investigator or co-investigator (where the responsibility has been delegated by the Principal Investigator as captured on the Site Signature and Delegation Log) to obtain informed consent/eConsent for each patient before performing any trial related procedure.

It is not expected that patients considered for enrollment in this study will have the capacity to consent to take part due to the nature of the underlying disease process, as they will be under sedation and will be mechanically ventilated. As a result of this, the study information sheet will be made available to a personal legal representative (PerLR);

The personal legal representative will be fully informed of all aspects of the trial and the potential risks. The following will be emphasised:

- Participation in the study is entirely voluntary.
- Refusal to participate involves no penalty or loss of medical benefits.
- The patient may withdraw from the study at any time.
- The personal legal representative is free to ask questions at any time to allow him or her to understand the purpose of the study and the procedures involved.
- There may be no direct benefit from participation.

- The aims of the study and all tests to be carried out will be explained. The personal legal representative will be given the opportunity to ask about details of the trial and will then have time to consider whether to participate or not.
- The risks of participating in the study will be fully explained.

If the personal legal representative decides that they wish for the patient to participate, they will sign and date the legal representative declaration form and will be given a copy. The original legal representative declaration form will be stored as source documentation. These forms will also be signed and dated by the Investigator.

In cases where it is not possible for a personal legal representative to be present in the hospital due to local visitor restrictions, the study can be discussed with the patient's personal legal representative over the telephone. A copy of the legal representative declaration form can be sent to the personal legal representative via email or online declaration form and returned to the study team.

There will also be an option for the personal legal representative to give assent electronically via a secure website. The telephone discussion will be documented in the patient's medical notes alongside a copy of the legal representative declaration form.

A professional legal representative (usually a consultant not directly involved in the study) will be asked to counter sign the legal representative telephone declaration form in situations where personal legal representative assent has taken place remotely and it has not been possible for the declaration form to be completed and signed by the personal legal representative.

COVID-19 patients intubated are at risk of rapid deterioration and given the urgent need to initiate treatment on the patients and the potential benefit of starting the treatment within 24 hours after intubating. If it is not possible to approach a personal legal representative for assent, a professional legal representative will decide regarding study participation on the patient's behalf. If the professional legal representative agrees for the patient to take part, they will sign a professional legal representative declaration form; the form will also be signed by a member of the study team (delegated by PI to take consent). The discussion will be documented in the patient's medical notes alongside a copy of the professional legal representative declaration form.

Personal or professional legal representative will be given sufficient time to review the information provided and ask questions before signing the assent / declaration form. Only members of the research team who have undergone the appropriate GCP training will be able to seek agreement from potential patients' personal or professional legal representative.

Once participants have regained capacity, they will be asked to consent to their continued participation in the study. The study rationale and conduct will be explained to them outlining any interventions which have already been undertaken as well as those that are still required.

Details of the informed consent or assent discussion will be recorded in the patient's medical record. This will include the date consent was given, with the name of the trial and the version number of the Participant Information Sheet and Informed Consent Form. Throughout the trial the patient or personal legal representative should have the opportunity to ask questions about the trial and any new information that may be relevant to the patient's continued participation should be shared with them in a timeously. On occasion, it may be necessary to re-consent the patient or personal / professional legal representative, for example if new information becomes available or an amendment is made to the protocol that might affect the patient's participation in the trial. In this case the process above will be followed and the patient's right to withdraw from the trial respected.

A flow chart summarizing the consent and assent processes is included below.

Assent and consent flow chart

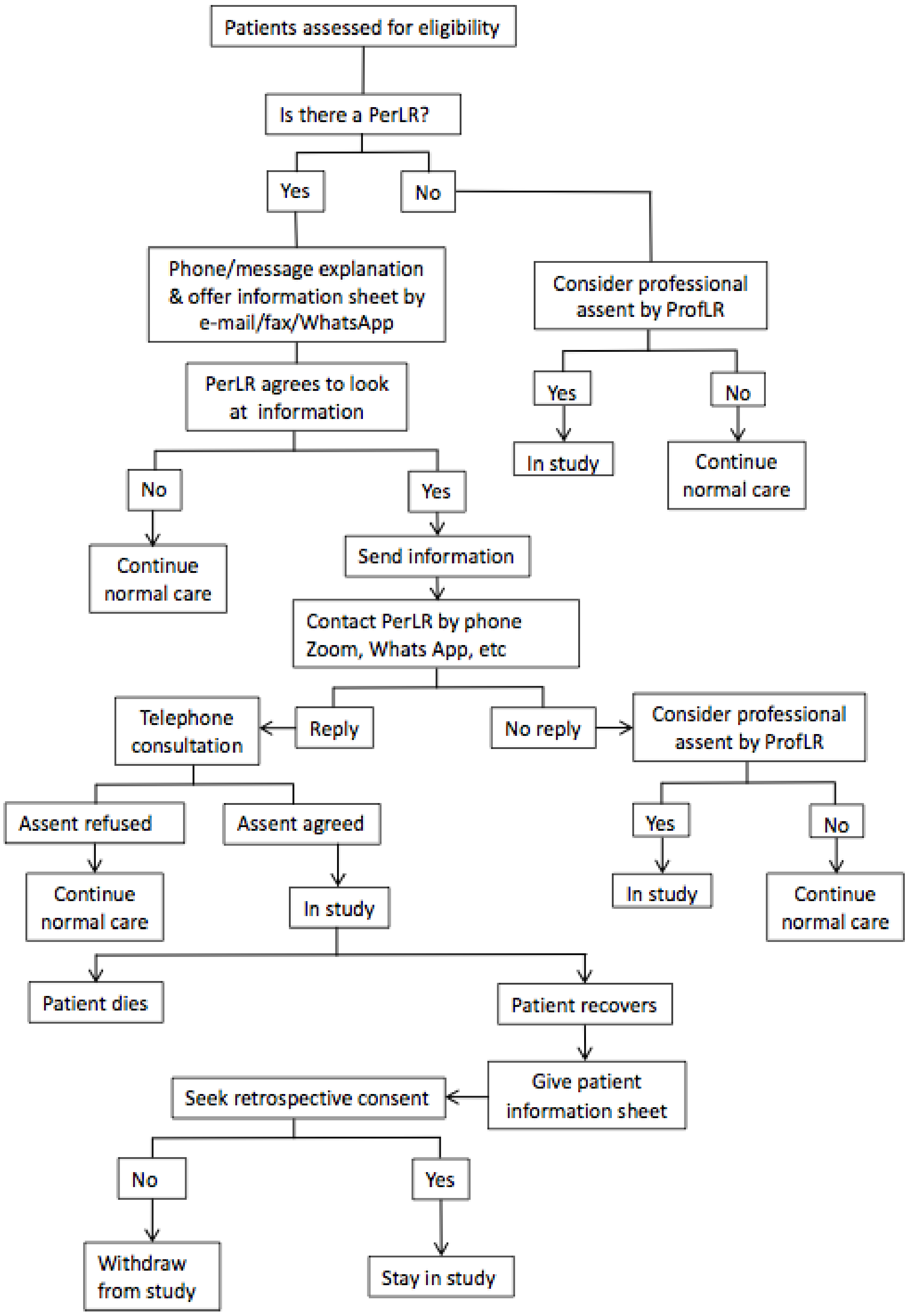

7.2.1 Additional consent provisions for collection and use of participant data and biological specimens in ancillary studies, if applicable

Participants will be asked to consent for the use of their samples in future research. Consent for this is optional. If the participant chooses not to consent to this their samples will be destroyed at the end of the study. Any samples stored for future research will be anonymised. Should a participant choose to withdraw from the study prior to completion, they will be able to withdraw their consent for their samples to be stored and the samples will then be destroyed.

7.3 The randomisation scheme

Patients will be randomised to 3:2 for the treatment and control arms using ALEA Randomisation. ALEA Randomisation is an internet-based service provided by FormsVision BV that supports online patient registration and randomisation and is fully integrated with ALEA eCRF. ALEA Randomisation has been built as a series of components, in order to support interoperability and customised extensions. The components make use of a common standard representation of data and metadata: The Operational Data Model of CDISC. Within ALEA Randomisation, the components share a database for storing and retrieving information about the trial, and a separate database for storing and retrieving patient data. ALEA Randomisation supports the following methods of randomisation for healthcare trials. It supports trials with any number of treatment groups, and randomisation ratios other than 1:1. In this study we will be using the block randomisation schema.

The following series of numbers will comprise the subject identifier:

- Three alpha characters – site code – UHS and UCH

- 2-digit subject number (e.g. 03)

Will be allocated by the site, reflecting the order of consent to the study within the site. For example, the third subject who signed an informed consent form at study UHS will be identified as Subject UHS-03.

The enrolment number will be added to the consent form at the site.

7.4 Blinding

N/A. This is an open-label study.

7.5 Baseline data

Baseline data on vital sign measurement and ventilation parameters will be recorded from data already generated in the intensive care unit. These values will be obtained closest to prior to the time of study initiation. Prior to administration of the surfactant a TA sample and EDTA and Transfix blood samples will be obtained from the patient and a chest ultrasound will be performed before and after.

<u>Demographic Information</u>

1. Patient demographics
2. Age, gender, ethnicity, height, weight, predicted body weight
3. Clinical frailty score (very fit, Well, managing well, vulnerable, mildly frail, moderately frail, severely frail, very severely frail and terminally ill)
4. Date/time of hospital admission
5. Date/time of ICU admission
6. Date/time of mechanical ventilation
7. List of medications on admission to hospital
8. List of medication on admission to ICU
9. Co-morbidities

a. Hypertension
b. Diabetes
c. Chronic kidney disease
d. Chronic respiratory diseases (inc. Asthma, COPD)
e. Chronic cardiac disease
f. Cardiovascular disease (inc. Stroke/TIA, STEMI/NSTEMI/CABG/Heart failure)
g. Cancer (active or treatment within a year)
h. Chronic liver disease (inc. ALD, NASH, cirrhosis, liver failure)
i. Immunosuppression
j. Other medical history or co-morbidities

APACHE 2 Score at ICU admission
Date/time collected
History severe organ failure or immunocompromise (heart failure class IV, cirrhosis, chronic lung disease, or dialysis dependent) (Yes/No), age, temperature, MAP, pH, heart rate, respiratory rate, sodium, potassium, creatinine, haematocrit, WBC, GCS, PaO_2, FiO_2 and acute kidney injury (Yes/No/unknown/N/A)

SOFA score calculations
Date/time collected
Cardiovascular score, respiratory score, coagulation score, hepatic score, renal score and CNS score

Physiology at baseline (closest to prior to study initiation)
1. Pulse, blood pressure, oxygen saturation and body temperature
2. Ventilator setting (date/time of measurements)
   Mode (mandatory, spontaneous, mixed), PEEP, tidal volumes, compliance, mean airway pressure, peak airway pressure, plateau pressure, I: E ratio, respiratory rate, use of nitric oxide, supine or prone
3. Oxygenation
   PaO_2, FiO_2, oxygen saturations, pH, CO_2, lactate
4. Blood markers:
   Haemoglobin, haematocrit, WBC, neutrophil, lymphocyte, eosinophil, platelet, INR, PT, ALT, ALP, bilirubin, AST, sodium, potassium, urea, creatinine, glucose, CRP, PCT, LDH, D-dimer, triglycerides, troponin, ferritin and CK
5. Radiological Markers
   CXR- are infiltrates present? Number of quadrants? and CXR report
6. USS of lungs
7. The need for renal replacement therapy
8. The need for cardiovascular support
9. The use of adjunctive therapies
   (E.g. Antivirals, antibiotics, steroids, list of sedation medications, list of Inotrope/vasopressor medications)

7.7 Trial assessments

Vital Signs & Ventilation Parameters

Pulse, blood pressure, body temperature, oxygen saturations, PaO_2, FiO_2, PEEP requirements and tidal volumes will be recorded at the time points indicated in the schedule of events (appendix 1). These will be measured constantly whilst the patient is managed by the intensive care team.

Vital signs and ventilation requirements will be recorded at baseline. During surfactant administration, the above vital sign and ventilation parameter measurements will be recorded in between the administration of each vial. Following each dose, these will then be recorded hourly until the administration of the next dose and this will then be repeated. Following administration of the final dose, vital sign and ventilation parameter measurements will be recorded at 30 hours post first dose and then 6 hourly until 48 hours post first dose.

Measurements will then be taken daily during the follow up period.

Chest Ultrasound

A chest ultrasound will be conducted at the time points indicated in the schedule of events (appendix 1). For patients randomised to the treatment arm, a chest ultrasound will also be performed before and after each dose of surfactant. The chest ultrasound will be carried out through use of the Butterfly IQ ultrasound device. Images will be stored in a secure database.

Administration of Nebuliser

The AeroFact-COVID™(AF-COVID) is an investigational drug/device combination product.

(Details of functioning and dosing are included in section 8 of this Protocol and in the IB and Device Technical File)

Infusion of *methyl*-D_9-choline chloride

Deuterated *methyl*-D_9-choline chloride will be dissolved in water at 10 mg/ml and infused at a dose of 3 mg/kg body weight over 3 hours, initiated during the stabilization period between intubation and administration of the first dose of surfactant. This timing is chosen to avoid conflict with time-consuming process of surfactant nebulisation. *Methyl*-D_9-choline incorporation into lung surfactant dipalmitoyl phosphatidylcholine (DPPC), the major surface-active component, will measure surfactant phospholipid synthesis. It will answer several mechanistic questions. These include:

2.) Is surfactant therapy associated with an earlier restoration of surfactant synthesis, indicating recovery?

3.) Does the synthesis analysis confirm a reduced number of ATII cells? Can incorporation results provide an estimate of the efficiency of nebulized surfactant delivery to the peripheral lung?

The MHRA have confirmed that *methyl*-D_9-choline chloride is not an IMP. A metabolic pathway for *methyl*-D_9-choline incorporation into PC is shown below, together with an example of the diagnostic mass spectrometry scans used for detection and quantification of unlabelled and deuterium-labelled PC.

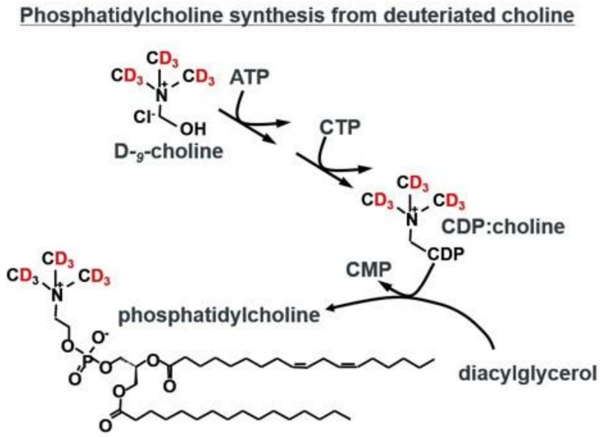

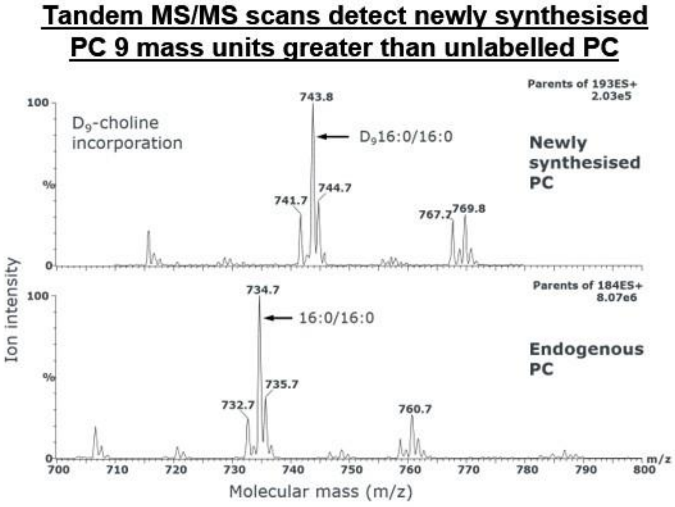

Sample collection

Samples to be taken for a combination of trial assessment and mechanistic studies are:

Blood Sample Collection

EDTA (10 ml) and Transfix (9 ml) blood samples will be taken at 0, 8, 24, 48 and 72 hours (while hospitalised). Blood samples will be collected immediately before administration of the first dose (t=0h), second (t=8h) and third (t=24h) doses of surfactant.

Arterial blood gasses (~1 ml) will be taken at intervals for the clinical trial at 0, 1, 2, 3, 6, 8, 9, 10, 11, 14, 18, 24, 25, 26, 27, 30, 36 hours. Results will be made available to clinicians and will largely replace the requirement for samples to be taken for routine clinical care which would normally require 6-12 blood gasses within 24 hours.

Urine pregnancy test

Urine will be tested for β HCG at screening for female volunteers of childbearing potential. As patients will not have the capacity to confirm their medical history in relation to their childbearing potential and this information may not be available in their medical notes, investigator discretion will be used to confirm whether pregnancy testing is required.

Closed suction tracheal aspirate samples

TA samples will be taken at 0, 8, 16, 24, 48 and 72 hours. The t=0-hour sample will additionally provide an estimate of baseline surfactant composition and concentration. The subsequent samples will be used for the mechanistic studies outlined above (section 2) and will assess the efficacy of surfactant administration. Baseline TA samples will be collected immediately before administration of the first dose of surfactant (t=0h). Samples will then be collected immediately before the second (t=8h) and third (t=24h) doses of surfactant and at 16, 48 and 72 hours thereafter if the patient is still intubated.

Additional TA samples will be taken for diagnostic PCR for SARS-CoV-2 on Days 3 (72 hrs), 5, 10, 15 and 28 (if still hospitalised).

7.8 Long term follow-up assessments

Patients will be monitored constantly as part of their routine clinical care in the intensive care unit. For the purpose of the study, follow up data will be recorded hourly from the administration of the last dose

of surfactant (24 hours) until 32 hours post first dose, 4 hourly until 48 hours post first dose and then daily until day 28, or until the time that the patient is discharged from hospital if this takes place first. A chest ultrasound will be performed daily until this time.

There is no anticipated risk of losing patients to follow up given that they will be hospitalised. Patients may choose to withdraw from the study when they re-gain the capacity to consent for themselves. In these cases, no additional date will be collected. Data can be collected retrospectively by the research team as intensive care records will remain available.

7.9 Qualitative assessments

This is not directly applicable to this study.

7.10 Withdrawal criteria

Withdrawal from the study will only occur if the patient or personal legal representative withdraws consent or safety event in the opinion of the investigators.

7.11 Storage and analysis of clinical samples

All samples will be collected into pre-labelled containers supplied in kits for individual patients by the clinical trial team, will be double bagged and transferred in a closed container between the ICU and the processing laboratory.

a. Baseline samples of lung TA samples will be collected up to 30 minutes before administration of the first dose of surfactant (t=0h). Samples will then be collected up to 15 minutes before the second (t=8h) and third (t=24h) doses of surfactant, at 16 hours and at 48 and 72 hours thereafter if the patient is still intubated. The TA samples will consist of blind suctioning by a cannula introduced through a port into the endotracheal tube. A volume of 20-50 ml saline will be administered, with an estimated recovery of 8-10 ml. This procedure will not interrupt the ventilator circuit and will not result in desaturation. TA samples will be taken into pre-labelled tissue culture (Falcon) tubes and stored at +4°C in the ICU sample fridge until transfer to the laboratory for processing. Transfer to the laboratory should be as rapid as possible, with a goal of under 2 hrs.
b. Blood samples (10 ml) will be collected into EDTA vacutainers and an additional sample (9) ml will be taken and fixed in Transfix tubes. Samples will be stored at +4°C until transfer to the laboratory for processing.
c. TA samples will be centrifuged at 400 x g x10 mins and the supernatant removed. Cell pellet differential white cell distributions will use cytospins after fixation. Aliquots for the different assays will be prepared from the supernatant. The aliquot for lipid analysis (800 µl) will be added to ice-cold methanol in glass vials and stored at -20°C until analysis. One aliquot of supernatant (1.5 ml) will be taken for surfactant protein analysis with the remainder stored in aliquots (1 ml) in cryovials. All cryovial samples will be stored at -80±5°C until analysis.
d. Blood samples will be centrifuged at 1200 x g x 10 mins. Two aliquots of plasma (200 µl) will be added to glass vials containing ice-cold methanol (2 ml) and stored at -20°C until lipid analysis. The remaining plasma will be stored in aliquots (500 µl) in cryovials at -80±5°C. Blood samples in Transfix tubes will be stored at +4°C.

All samples will be stored within sealed storage containers in identified secure refrigerators and freezers. Temperature of the -80°C freezers will checked daily by a data logger.

There will be a schedule of courier transfer between the three analytical sites. As Transfix tubes can be stored for up to 7 days before analysis, the 6 samples from each patient will be stored together and sent to Manchester in individual patient batches. Sample transfers between the London and Southampton sites will be scheduled for completion of the trial.

All aliquots of TA and blood plasma samples that remain after the analytical procedures will be stored at -80±5ºC until a decision to gift samples for future research is made either by patients after recovery or by a personal legal representative for patients who have died. If such permission is refused, samples concerned will be destroyed according to the normal trust guideline.

Virus will be destroyed where practicable in the samples for analysis. This will be achieved for the lipid analyses during extraction by adding samples to methanol, by UV irradiation for the surfactant protein analysis and by fixation for the inflammatory cell characterisation. All procedures on samples with live virus will be undertaken in category 2 or 3 facilities as appropriate and related SOPs will be supplied. They will be:

1. Blind closed suctions TA samples, giving 20-50 ml saline with typical recovery of 8-10 ml
2. EDTA blood samples.

Analyses to be undertaken by laboratories at Southampton, London and Manchester on samples from both surfactant and control groups are:

1. **Surfactant lipid composition and concentration (University of Southampton)**

   Mass spectrometric analysis of molecular species compositions of phosphatidylcholine (PC), phosphatidylglycerol (PG) and phosphatidylinositol (PI) in small volume TA samples. We expect reduced surfactant concentration and altered composition in baseline samples taken immediately before surfactant administration. These would both change after surfactant is given, and we might be able to assess the ratio of exogenous to endogenous surfactant from these two different compositions. Analysis of sequential samples (8 hrs, 16 hrs, 24 hrs, 48 hrs and 72 hrs if still intubated) will provide an indication of exogenous surfactant turnover.

2. **Surfactant function (Imperial College, London)**

   Surfactant function will be determined by measuring surfactant adsorption to the air: liquid interface in a Langmuir trough. We will measure the tensioactive properties of lung surfactant from patients' TA. We will investigate whether isolated surfactant will have compromised function by the inhibition of plasma proteins, and whether the function can be recovered after administration of supplemented surfactant. Maximum reduction of the surface tension over continuous compression: expansion cycles models the physiological breathing cycles of intubated patients and will be carried on a captive bubble surfactometer. Adsorption analysis of the equilibrium surface tension, together with kinetics of the process, will explore inhibition processes in surfactant function.

3. **Immunogical assessment (University College London and University of Manchester)**

   Immune cellular components will be profiled to depth and soluble fluids for soluble mediators including those that imply a bacterial or fungal co-infection. The volume needed would depend on the concentration of the cellular component. Fixed blood will be split into 3 and then to each, a cocktail of antibodies will be added to enumerate all T cell subsets and markers of their subtype and activation. B cell subsets and their antibody isotype will also be investigated. In a separate panel we will identify neutrophils, eosinophils, dendritic cells, monocytes and macrophages. These panels have already been validated on blood samples from patients with COVID-19 in Manchester. The proportions of cells identified will be compared to normal ranges

within each age group. We will be specifically looking at the neutrophil to lymphocyte ratio that is described to be inverted in patients with the disease.

The level of the immunomodulatory surfactant protein D (SP-D) in serum has shown to be a promising biomarker for lung inflammation (and hence low levels in TA), and we will quantify the level of SP-D in both TA and plasma using our established ELISA. We will also quantify the level of C-reactive protein in TA as a biomarker for blood contamination of surfactant together with total protein concentration. We have seen that an inflammatory response to infections can results in specific degradation dependent on the infectious agent. We will analyse the degradation of SP-D and its sister protein SP-A using SDS-PAGE and Western Blot analysis to evaluate if COVID-19 infection results in degradation.

4. **Oxidative Stress/Redox Measurements**
   The extent of local and systemic oxidative stress with associated modifications of surfactant composition, alterations in cell signalling (due to interference with nitric oxide and hydrogen sulfide-related cell function) and shifts in redox status following the increased production of reactive oxygen species (downstream effects of the “cytokine storm”) induced by SARS-CoV-2 will be characterised in aliquots of pulmonary secretions and blood. 0.5 mL of bronchial fluid and a total of 1.5 mL EDTA plasma will be used for these analyses. Plasma will be divided into three 500 µL cryovial aliquots directly after centrifugation, frozen in liquid nitrogen and kept at -20ºC until analysis.

   Oxidative stress, nitric oxide, hydrogen sulfide and other redox related metabolites and products of ‘reactive species’ interactions will be quantified by an array of analytical platforms including ELISA, gas-phase chemiluminescence, HPLC, IC-MS, and LC-MS/MS to determine the following readouts: 8-isoprostanes, malondialdehyde (TBARS), nitrite, nitrate, total nitroso species, thiosulfate, sulfate, total free thiols, free and bound low-molecular weight thiols (including cysteine, homocysteine, glutathione), sulfide and polysulfide species.

5. **Surfactant phosphatidylcholine synthesis**

   Infusion of stable isotope-labelled choline (methyl-D9-choline chloride) IV will provide a measure of surfactant PC synthesis and hence function of ATII cells. This would be incorporated into the phospholipid analysis. The concentration of *methyl*-D_9-PC molecular species will determine if surfactant therapy enhances synthesis and secretion of endogenous surfactant PC and administration of the second and third doses of unlabelled exogenous surfactant at t=8 and 24 hours will permit calculation of endogenous and exogenous surfactant pool sizes by dilution of the endogenously labelled surfactant. The only additional workload here would be IV infusion of *methyl*-D_9-choline choline chloride over 3 hours after the patient is intubated and before the first surfactant administration. Patients in the control group will receive *methyl*-D_9-choline at a time equivalent to surfactant administration in the treatment group, followed by identical sample collection.

7.12 End of trial

The end of study is defined as the completion of the testing of samples, to be achieved no later than 3 months after the date of the last visit of the last patient.

8 TRIAL TREATMENTS

8.1 Name and description of investigational medicinal product(s) and device

The AeroFact-COVID™(AF-COVID) is an investigational drug/device combination product. The proposed indication for the AeroFact-COVID™ combination product is for the treatment of COVID-19 related Adult Respiratory Distress Syndrome (ARDS) in intubated, mechanically ventilated adults.

IMP: Bovactant (Alveofact®) is a lyophilised bovine lung surfactant presented as a lyophilized powder in 108 mg vials and reconstituted to 45 mg/mL in buffer supplied in a prefilled syringe.

Device: The nebulizer, AeroFact-COVID™ Device, is an investigational device based on the Aerogen® Solo, the market-leading vibrating mesh nebulizer for use in critical care and manufactured in Ireland by Aerogen®. It is approved in more than 75 countries and sold in excess of 2 million units in 2019.

AF-COVID combination product consists of three basic components: (i) drug, a lyophilized SFRI 1 bovine-origin surfactant and diluent for reconstitution in a vial with a reconstitution kit (marketed as Alveofact®1 by Lyomark GmbH (Germany)); (ii) reusable components: a Controller, a Synchronizer, AC/DC Adaptors and Cables; and (iii) disposable, single patient use nebulizer and respiration sensor.

Further information on Alveofact and AeroFact-COVID Device can be obtained from the Investigation Brochure and the Device Technical File.

8.2 Regulatory status of the drug

Bovactant was developed by Boehringer Ingelheim. In 1989, it received regulatory approval in Germany for prevention of RDS based on 8 clinical studies (detailed summary in included in the Investigator Brochure: Appendix II and repots on file at Lyomark Pharma and Aerogen Pharma). The regulatory approval was renewed on 9 September 2004.

In 2005 it was acquired by the current manufacturer, Lyomark Pharma (Oberhaching, DE). Since its original approval in Germany, SF-RI 1 (under the marketing name of Alveofact) has been approved in six European countries and in 24 countries outside the EU. The legal manufacturer of Alveofact, following post-marketing requirements, is following the safety profile and according to their most current Post- Marketing report, there are no safety trends or concerns based on self-reporting from centres using the drug.

8.3 Regulatory Status of Device

AeroFact-COVID™ is a non-CE marked device. AeroFact-COVID replaces the standard vibrating mesh plate with an alternative of novel architecture but identical material construction as the commercially available Aerogen® Solo nebulizer (K133360, K103635 and K070642, Aerogen Ltd.) This novel “PDAP” mesh is capable of effectively nebulizing pulmonary surfactant, which is otherwise challenging to aerosolize, and generates smaller droplets 1 to 4 microns in size which enhance deep lung delivery. The nebulizer is driven by a small, pole-mounted controller (mains with battery back-up; UK version available), and can be fitted into an endotracheal tube adapter, a mouthpiece or a face mask for treatment of invasively ventilated or spontaneously breathing patients.
The device is a combination of CE-Marked devices and Investigational Devices, with reusable or disposable components:

Reusable Components:
Areogen Nebulizer Cable – CE Marked
AC/DC Adapter – CE Marked
Aerogen Controller Bracket – CE-Marked
AF–COVID Controller – Investigational Device
AF–COVID Synchronizer – Investigational Device
Sensirion Flow Sensor SFM3300 Cable – Investigational Device

Disposable Components:

AF-COVID Nebulizer: Areogen Solo Nebulizer Housing – CE-Marked
AF-COVID Nebulizer: Areogen 15mm T-Piece Connector to the Endotracheal Tube – CE-Marked
AF-COVID Nebulizer: PDAP Mesh – Investigational Device
Sensiron Flow Sensor SFM3300 – Investigational Device

8.4 Product Characteristics

Bovactant (Alveofact®)

The finished dosage form is a SF-RI 1 lyophilizate that contains no additional excipients and hence has the same chemical composition as the drug substance. The labelled dose in each Type 1, clear glass, 6R vial is 108 mg. The closure is a gray butyl rubber, fluoropolymer-coated, 13 mm stopper with an aluminum/propylene, blue, 13 mm flip-off seal. The current manufacturing facility is BAG Health Care (BAG) GmbH, Amtsgerichtstrasse 1-5, 35423 Lich, Germany.

As a natural animal derived surfactant preparation, absolute compositional and structural formulae cannot be provided as in the case of a synthetic preparation. It is isolated from bovine lung lavage and, by weight, is a mixture of:

- Phospholipids, approximately:
  - 75% phosphatidylcholine (PC, also known as lecithin)
  - 13% phosphatidylglycerol (PG)
  - 3% phosphatidylethanolamine (PE)
  - 1% phosphatidylinositol (PI)
  - 1% sphingomyelin
- 5% Cholesterol
- 1% lipid-soluble surfactant-associated proteins, SP-B and SP-C
- Very low levels of free fatty acid, lyso-phosphatidylcholine and water
- 0.3% calcium

AeroFact-COVID Device

The AeroFact-COVID™ Nebulizer is a vibrating mesh nebulizer that is operated electronically by a signal generated continuously with the AF-COVID Controller. The drug is delivered to the mesh nebulizer directly by dispensing into the medication reservoir.

The system generates aerosol with a droplet size distribution smaller than conventional nebulizer systems. Particle size distribution <5 μm mass median aerodynamic diameter (MMAD) was chosen to minimise deposition in tubing and airways, to maximize the potential dose reaching the lungs and to minimise interpatient variability. The nebulizer is positioned between the ventilator circuit and the endotracheal tube or artificial airway, ensuring direct patient drug delivery to minimise drug loss and drug emissions. The system is designed to generate aerosol in synchrony with delivered breaths

during mechanical ventilation, rather than continuously. The delivery rates are up to 0.3 mL/min when used with SF-RI 1 surfactant suspension. The mean delivery rate during breath-synchronized operation is slower than this due to interruption in synchrony with typical Inspiration: Expiration (I: E) ratios.

The AF-COVID system is based on CE-marked components with modifications improving its performance to answer the current clinical need for delivery of aerosolized surfactant into the lungs of adult patients infected with COVID-19 and showing ARDS symptoms.

8.4 Drug storage and supply

Both the vials of lyophilized SF-RI 1 and pre-filled sterile syringe containing 0.45% saline should not be stored above 30ºC (86ºF) and should not be allowed to freeze. Once the drug product has been reconstituted with 0.45% saline, the resulting suspension can be stored up to 4 hours at 2-8°C or room temperature. In such cases, the vial containing the suspension should be gently inverted 5 times before use. Study medication will be stored in a secure, controlled-access location at the study sites.

Any deviations from the storage conditions should be reported immediately to the Sponsor, and the study medication should be placed in quarantine and not used until authorisation has been given by the Sponsor to do so.

The storage conditions of the re-usable controller and the single-patient, single use disposable, drug delivery circuit and breath sensor are:

Temperature: -20 to +60°C
Relative humidity: 15 – 95 %RH
Atmospheric pressure 450 – 1100 kPa

For the AF-COVID clinical development program, SF RI 1 will be sourced from Lyomark Pharma GmbH and supplied to the clinical study sites as a lyophilized formulation of 108 mg, along with the required diluent for reconstitution, and labelled for clinical investigational use only.

<u>Storage conditions for the device</u>

The storage conditions of the re-usable controller and the single-patient, single use disposable, drug delivery circuit and breath sensor are:
Temperature: -20 to +60°C
Relative humidity: 15 – 95 %RH
Atmospheric pressure 450 – 1100 kPa

Aerogen Pharma will provide the AeroFact AF-COVID investigational product.

Both IMPs and devices will be sent directly to each site Pharmacy from the relevant supplier. Pharmacy will be responsible for recording receipt of product and devices and for dispensing to the Investigator for trial use.

The Investigator (or designee) is responsible for ensuring study medication and device accountability, including administration records and returns. The Sponsor will provide study medication accountability forms for this purpose.

Unused study medication or devices must not be discarded without authority from the Sponsor or used for any purpose other than the present study.

8.5 Preparation and labelling of Investigational Medicinal Product

Alveofact® lyophilized vials (108 mg) and pre-filled sterile syringe containing 0.45% saline come ready to be mixed. Once the drug product has been reconstituted with 0.45% saline, the resulting suspension can be stored up to 4 hours at 2-8°C or room temperature. The vials and syringes will be labelled in accordance with regulatory requirements.

8.6 Preparation and labelling of Investigational devices

The contents of the label will be in accordance with all applicable regulatory requirements.
In App 3 of the Device Technical File, are the Labels for AF-COVID Devices:
1.- Investigational Reusable controllers:
a) AF-COVID Controller Device Label
b) AF-COVID Synchronizer Device Label
c) AF-COVID Controller & AF-COVID Synchronizer Kit Box Label
2.- Investigational Disposable Components:
a) AF-COVID Nebulizer Device Label
b) AF-COVID Nebulizer Pouch and Box Label
3. CE Marked Disposable Components:
a) 15mm T-Piece

8.7 Dosage schedules

Sequential vials of 108 mg Alveofact® in 2.2 mL buffer will be administered by nebulizer at 5-minute intervals with observation of vital signs, changes in oxygen saturation, FiO_2 and Mean Airway Pressure. After each administration, 2 drops of normal saline will be nebulized, and the next vial administered. After administration of the first dose, dosing of Alveofact® will pause and vital signs and ventilation parameters will be assessed over the next 8 hours. If no grade 3 or grade 4 adverse events attributable to surfactant administration occur, another dose of Alveofact® will be administered and vital signs and ventilation parameters will be assessed over the next 16 hours. Again, if no grade 3 or grade 4 adverse events attributable to surfactant administration occur; a final dose of Alveofact® will be administered.

The dosing schedule for each group is displayed in the table below.

Group	0 Hours	8 Hours	24 Hours
1	10 x 108 mg vials	10 x 108 mg vials	10 x 108 mg vials
2	10 x 108 mg vials	10 x 108 mg vials	30 x 108 mg vials
3	10 x 108 mg vials	30 x 108 mg vials	30 x 108 mg vials
4	30 x 108 mg vials	30 x 108 mg vials	30 x 108 mg vials

If a dose time point is missed it should be administered as soon as it is possible. The investigator or delegate will assess the clinical stability of the patient prior to administering the dose or following a time point being missed or treatment being suspended.

8.7 Dosage modifications

In an event when the administration of 30 vials is not practical, doses may drop to 20 or 10 vials, depending on feasibility. In the event clear efficacy is observed in cohort 1, doses may drop below 10 vials or dosing times may be reduced.

Progression and dosing modifications of subsequent cohorts will be decided by teleconference of the trial steering committee. Any safety concerns would be referred to the independent DSMB on completion of dosing of each cohort of 3 patients + 24 hours to enable collection of physiological outcomes.

The DSMB will actively monitor emerging safety and efficacy data to make recommendations about early study closure or the initiation of a larger randomised controlled clinical trial.

The dose administered cannot be modified due to patient / personal legal representative request.

Given the severity of the illness in ventilated Covid-19 patients, safety data will be interpreted with caution and will not be formally compared between active intervention and control groups.

- Dosing will be stopped in individual patients upon persistent deterioration of oxygenation during or after surfactant administration
- Study stopping rules for futility or efficacy are provided in the statistical analysis section.
- The protocol team will review emerging AE/SAE/SADEs data daily. If 3 or more patients develop the same SAE after administration of study drug, the study will be paused pending review by the DSMB. The study may also pause at the discretion of the Principal Investigator if there are a significant number of unexpected AEs/SAEs/SADEs and referred to the DSMB for review.
- The DSMB will formally review the safety data after every three subjects and is available for ad hoc reviews for other safety concerns as noted above.

8.8 Known drug reactions and interaction with other therapies

None known.

8.9 Concomitant medication

Concomitant medication administered as part of routine clinical care whilst the patient is hospitalised will be recorded in the eCRF. This data will be available in the patients' medical records. No routine concomitant medications will be withheld as a result of participation in this study.

8.10 Trial restrictions

There are no restrictions in relation to dietary requirements or contraception in this study. Patients will be hospitalised, and their care will be managed by the direct clinical care team in the intensive care unit.

8.11 Assessment of compliance with treatment

Compliance will be assessed by the investigator or delegate as part of the clinical monitoring required for the study (as shown in the schedule of events, appendix 1). Vital signs and ventilator parameters will be reviewed on an ongoing basis. Non-compliance with treatment will be recorded in the eCRF and patient medical notes. Non-compliance resulting in an AE, SAE or SUSAR will be recorded and

reported according to the information outlined in section 9.3. Follow up of non-compliance related adverse reactions will be carried out in line with section 9.9.

9 PHARMACOVIGILANCE

9.1 Definitions

The following definitions have been adapted from Directive 2001/20/EC, ICH-GCP E6 (r2), Directive 93/42/EC, ISO 14155: 2011 and MEDDEV 2.7/3:

Term	Definition
**Adverse Event (AE)**	Any untoward medical occurrence in a participant to whom a medicinal product has been administered, including occurrences which are not necessarily caused by or related to that product.
**Adverse Reaction (AR)**	An untoward and unintended response in a participant to an investigational medicinal product which is related to any dose administered to that participant.<br>The phrase "response to an investigational medicinal product" means that a causal relationship between a trial medication and an AE is at least a reasonable possibility, i.e. the relationship cannot be ruled out.<br>All cases judged by either the reporting medically qualified professional or the Sponsor as having a reasonable suspected causal relationship to the trial medication qualify as adverse reactions. It is important to note that this is entirely separate to the known side effects listed in the SmPC. It is specifically a temporal relationship between taking the drug, the half-life, and the time of the event or any valid alternative aetiology that would explain the event.
**Serious Adverse Event (SAE)**	A serious adverse event is any untoward medical occurrence that:<br>• results in death<br>• is life-threatening<br>• requires inpatient hospitalisation or prolongation of existing hospitalisation<br>• results in persistent or significant disability/incapacity<br>• consists of a congenital anomaly or birth defect<br>Other 'important medical events' may also be considered serious if they jeopardise the participant or require an intervention to prevent one of the above consequences.<br>NOTE: The term "life-threatening" in the definition of "serious" refers to an event in which the participant was at risk of death at the time of the event; it does not refer to an event which hypothetically might have caused death if it were more severe.
**Serious Adverse Reaction (SAR)**	An adverse event that is both serious and, in the opinion of the reporting Investigator, believed with reasonable probability to be due to one of the trial treatments, based on the information provided.
**Suspected Unexpected Serious Adverse Reaction**	A serious adverse reaction, the nature and severity of which is not consistent with the information about the medicinal product in

**(SUSAR)**	question set out in the reference safety information:<br>• in the case of a product with a marketing authorisation, this could be in the summary of product characteristics (SmPC) for that product, so long as it is being used within its licence. If it is being used off label an assessment of the SmPCs suitability will need to be undertaken.<br>• in the case of any other investigational medicinal product, in the investigator's brochure (IB) relating to the trial in question
**Adverse Event (AE)-(device)**	Any untoward medical occurrence, unintended disease or injury, or untoward clinical signs (including abnormal laboratory findings) in subjects, users or other persons, whether or not related to the investigational medical device<br><br>NOTE 1 This definition includes events related to the investigational medical device or the comparator.<br><br>NOTE 2 This definition includes events related to the procedures involved.<br><br>NOTE 3 For users or other persons, this definition is restricted to events related to investigational medical devices
**Adverse Device Effect (ADE)**	Adverse event related to the use of an investigational medical device.<br>NOTE 1 This definition includes adverse events resulting from insufficient or inadequate instructions for use, deployment, implantation, installation, or operation, or any malfunction of the investigational medical device.<br>NOTE 2 This definition includes any event resulting from use error or from intentional misuse of the investigational medical device.
**Serious Adverse Device Effect (SADE)**	Adverse device effect that has resulted in any of the consequences characteristic of a serious adverse event.
**Anticipated Serious Adverse Device Effect (ASADE)**	Anticipated serious adverse device effect (ASADE) is an effect which by its nature, incidence, severity or outcome has been identified in the risk analysis report or Investigator's Brochure.
**Unanticipated Serious Adverse Device Effect (USADE)**	Serious adverse device effect which by its nature, incidence, severity or outcome has not been identified in the current version of the risk analysis report or Investigator's Brochure.
**Device Deficiency (DD)**	Inadequacy of a medical device with respect to its identity, quality, durability, reliability, safety or performance<br>NOTE Device deficiencies include malfunctions, use errors, and inadequate labelling

NB: to avoid confusion or misunderstanding of the difference between the terms "serious" and "severe", the following note of clarification is provided: "Severe" is often used to describe intensity of a specific event, which may be of relatively minor medical significance. "Seriousness" is the regulatory definition supplied above.

9.2 Operational definitions for (S)AEs/(S)ADEs

AEs, SAEs, SUSARs, ADEs, SADEs and USADEs will be recorded and reported according to applicable trial regulations.

All adverse events that occur between informed consent and until 28 days post cessation of trial treatment must be recorded in the subject notes and in the trial CRFs.

Pre-existing conditions (as recorded at screening visit) do not qualify as adverse events unless, in the opinion of the treating clinician, have worsened since baseline.

The following pre-specified respiratory deteriorations or adverse events occurring during nebulisation and occurring within 48 hours will be recorded:

1. Increase in oxygen or ventilator requirements [increase in $Fi0_2$ ≥0.2 or more and or increase in PEEP of ≥5 or more to maintain the targeted oxygen saturation
2. Sustained deterioration in pulmonary ventilation variables >10% (increase in peak or mean airway pressures, decrease in tidal volume)
3. Any episodes of new cardiac arrhythmia
4. Sustained reduction in mean arterial blood pressure (MAP) of >10% or an increase in the vasopressor dose of Norepinephrine (0.1 mcg/kg/min), Epinephrine (0.1 mcg/kg/min) or the use of additional use of other inotropes (dopamine/dobutamine/milrinone) or vasopressors (vasopressin, terlipressin, phenylephrine)
5. Sustained increase in heart rate >20%
6. New bronchospasm requiring treatment
   Other respiratory deteriorations: Incidence of pneumothorax (evidence on imaging), pulmonary haemorrhage (clinical), acute lobar collapse (evidence on imaging).

All SAEs and Device Deficiencies (as per definitions) must be recorded and reported to the Sponsor (sponsor@uhs.nhs.uk) and CRO (safety@pharmexcel-cro.com) immediately/within 24hrs of the site becoming aware of the event.

9.3 Evaluation of Adverse Events

When an Adverse Event occurs, the investigator (PI or delegated clinician) is responsible for reviewing documentation related to the event. The investigator will record all relevant information on the eCRF and make an assessment on seriousness, causality and severity.

9.3.1 Assessment of Severity

The PI or delegated clinician must perform an assessment of severity for each AE according to the following categories.

Severity category	Definition of severity category
Mild<br>(Grade 1)	AE that is easily tolerated by the trial participant, causing minimal discomfort and not interfering with everyday activities
Moderate<br>(Grade 2)	AE that is sufficiently discomforting to interfere with normal everyday activities

Severe (Grade 3)	AE that prevents normal everyday activities

9.3.2 Assessment of Causality

The PI or delegated clinician must perform an evaluation of causality for each adverse event. The investigator will use clinical judgement to determine relationship to IMP, device or device deficiency.

Causality Category	Causality category definition
Definitely	Temporal relationship of the onset, relative to administration of the product, is reasonable and there is no other cause to explain the event, or a re-challenge (if feasible) is positive.
Probably	Temporal relationship of the onset of the event, relative to the administration of the product, is reasonable and the event is more likely explained by the product than any other cause
Possibly	Temporal relationship of the onset of the event, relative to administration of the product, is reasonable but the event could have been due to another, equally likely cause.
Unlikely	Temporal relationship of the onset of the event, relative to administration of the product, is likely to have another cause which can by itself explain the occurrence of the event.
Not Related	Temporal relationship of the onset of the event, relative to administration of the product, is not reasonable or another cause can by itself explain the occurrence of the event.

9.3.3 Assessment of Seriousness

Those AEs/ARs/ADEs meeting the definition of Serious (SAE) as per section 9.1 must also be assessed for expectedness as per the Instructions for Use for the device. Each SAE must be reported to the Sponsor as per section 9.2 and 9.4.

9.3.4 Assessment of Expectedness

Since there is no current clinical *experience with AeroFact-COVID combination product, there are* **no observed expected adverse reactions** *that can serve the investigators as guidance for reporting serious adverse events in the clinical study. Therefore,* **all treatment related Serious Adverse Events (SAEs) will be considered unexpected** *and will be reported to the authorities as per national requirements.*

9.4 Recording and reporting of SAEs, SARs, SUSARs, SADEs, USADEs and DD

Any **SAE** occurring from the time of **start of trial treatment** until 28 days post cessation of trial treatment must be recorded on the combined IMP/Device SAE Report Form and Device Deficiency form (if SAE is related to DD) and sent to the Sponsor (sponsor@uhs.nhs.uk) and CRO (safety@pharmexcel-cro.com) **immediately or within 24 hours** of the research staff becoming aware of the event.

Any change of condition or other follow-up information should be sent to the Sponsor and CRO as soon as it is available or at least within 24 hours of the information becoming available. Events will be followed up until the event has resolved or a final outcome has been reached.

All SAEs assigned by the PI or delegate (or following central review) as suspected to be related to IMP-treatment are deemed unexpected, will be classified as SUSARs and will be subject to expedited reporting to the Medicines and Healthcare Products Regulatory Agency (MHRA) medicines sector.

For all Device Deficiencies, the investigator needs to complete the DD form and sent to the Sponsor (sponsor@uhs.nhs.uk) and CRO (safety@pharmexcel-cro.com) **immediately or within 24 hours** of the research staff becoming aware of the event.

Please refer to the Safety Reporting Flowchart below.

IMP reporting pathway - SUSAR Reporting

The CRO, on behalf of the sponsor, will inform the MHRA and the REC of SUSARs within the required expedited reporting timescales (15 days for all SUSARs, unless resulting in death or is life threatening in which case 7 days, with a final report within a further 8 days (total 15)). The sponsor will also inform all Investigators concerned of relevant information about SUSARs that could adversely affect the safety of participants.

Device reporting pathways

All SAEs and DDs (meeting criteria below) will be reported to the MHRA devices sector, by the CRO, using the MEDDEV SAE reporting form (MEDDEV 2.7/3). Timelines outlined below;

<table>
<tr><th>Term</th><th>Reporter</th><th>Reported to</th><th>Reporting Timeline from awareness of the event</th></tr>
<tr><td rowspan="2">Device Deficiency (DD)</td><td>Investigator</td><td>Sponsor/CRO</td><td>Immediately/within 24 hrs of becoming aware of event.</td></tr>
<tr><td>Sponsor/CRO</td><td>MHRA</td><td>Immediately but no later than 2 calendar days if the event may have led to an SAE if;
suitable action had not taken
intervention had not been made
if circumstances had been less fortunate</td></tr>
<tr><td rowspan="2">Serious Adverse Event (SAE)</td><td>Investigator</td><td>Sponsor/CRO</td><td>Immediately/within 24 hrs of becoming aware of event.</td></tr>
<tr><td>Sponsor/CRO</td><td>MHRA</td><td>Immediately but not later than 2 calendar days of sponsor becoming aware of event if event indicates an imminent risk of death,</td></tr>
</table>

			serious injury, or serious illness and that requires prompt remedial action for other patients, users or persons, or a new finding to it.<br><br>For all other events Immediately but not later than 7 calendar days of sponsor becoming aware of event
Serious Adverse Event (USADE)	Investigator	Sponsor/CRO	Immediately/within 24 hrs of becoming aware of event.
	Sponsor/CRO	REC	Within 15 calendar days of becoming aware of the event
New findings/updates in relation to already reported events	Sponsor/CRO	MHRA	Immediately but not later than 7 calendar days of sponsor becoming aware of event

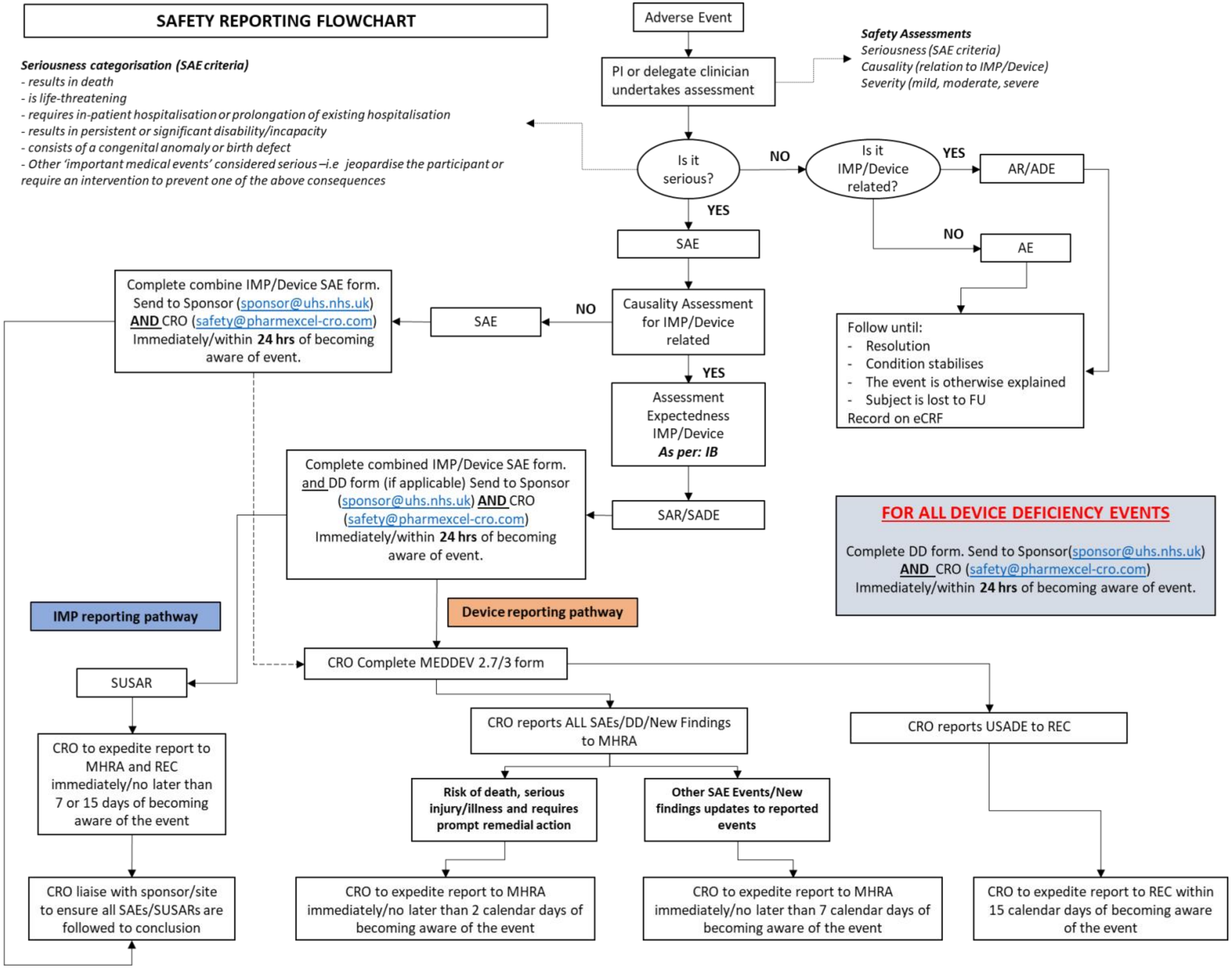
SAFETY REPORTING FLOWCHART
Seriousness categorisation (SAE criteria)
- results in death
- is life-threatening
- requires in-patient hospitalisation or prolongation of existing hospitalisation
- results in persistent or significant disability/incapacity
- consists of a congenital anomaly or birth defect
- Other 'important medical events' considered serious –i.e jeopardise the participant or require an intervention to prevent one of the above consequences
Adverse Event
PI or delegate clinician undertakes assessment
Safety Assessments
Seriousness (SAE criteria)
Causality (relation to IMP/Device)
Severity (mild, moderate, severe
Is it serious?
NO
Is it IMP/Device related?
YES
AR/ADE
NO
AE
YES
SAE
Follow until:
- Resolution
- Condition stabilises
- The event is otherwise explained
- Subject is lost to FU
Record on eCRF
Complete combine IMP/Device SAE form. Send to Sponsor (sponsor@uhs.nhs.uk) AND CRO (safety@pharmexcel-cro.com) Immediately/within 24 hrs of becoming aware of event.
SAE
NO
Causality Assessment for IMP/Device related
YES
Assessment Expectedness IMP/Device As per: IB
Complete combined IMP/Device SAE form. and DD form (if applicable) Send to Sponsor (sponsor@uhs.nhs.uk) AND CRO (safety@pharmexcel-cro.com) Immediately/within 24 hrs of becoming aware of event.
SAR/SADE
FOR ALL DEVICE DEFICIENCY EVENTS
Complete DD form. Send to Sponsor(sponsor@uhs.nhs.uk) AND CRO (safety@pharmexcel-cro.com) Immediately/within 24 hrs of becoming aware of event.
IMP reporting pathway
Device reporting pathway
CRO Complete MEDDEV 2.7/3 form
SUSAR
CRO reports ALL SAEs/DD/New Findings to MHRA
CRO reports USADE to REC
CRO to expedite report to MHRA and REC immediately/no later than 7 or 15 days of becoming aware of the event
Risk of death, serious injury/illness and requires prompt remedial action
Other SAE Events/New findings updates to reported events
CRO liaise with sponsor/site to ensure all SAEs/SUSARs are followed to conclusion
CRO to expedite report to MHRA immediately/no later than 2 calendar days of becoming aware of the event
CRO to expedite report to MHRA immediately/no later than 7 calendar days of becoming aware of the event
CRO to expedite report to REC within 15 calendar days of becoming aware of the event

9.4 Responsibilities

Principal Investigator (PI):

Checking for and assessing AEs when participants are admitted to hospital or return for follow-up.

1. Using medical judgement in assigning severity, seriousness, causality and expectedness as per approved IB for the trial.
2. Ensuring that all SAEs are recorded and reported to the sponsor within 24 hours of becoming aware of the event and provide further follow-up information as soon as available.
3. Ensuring that SAEs are chased with Sponsor if a record of receipt is not received within 2 working days of initial reporting.
4. Ensuring that AEs are recorded and reported to the sponsor in line with the requirements of the protocol.

Chief Investigator (CI) / delegate or independent clinical reviewer:

1. Clinical oversight of the safety of patients participating in the trial, including an ongoing review of the risk / benefit.
2. Using medical judgement in reviewing all SAEs (seriousness, causality and expectedness in line with approved IB.
3. Immediate review of all SUSARs/USADEs.
4. Review of specific SAEs /SARs/SADEs in accordance with the trial risk assessment and protocol as detailed in the Trial Monitoring Plan.
5. Assigning Medical Dictionary for Regulatory Activities (MedDRA) or Body System coding to all SAEs.
6. Preparing the clinical sections and final sign off of the Development Safety Update Report (DSUR).
7. Reviewing the quarterly safety reports to the device sector

Sponsor:

1. Central data collection and verification of all safety events according to the trial protocol onto a database – oversight, delegated to CRO
2. Reporting safety information to the CI, delegate or independent clinical reviewer for the ongoing assessment of the risk / benefit according to the Trial Monitoring Plan.
3. Reporting safety information to the independent oversight committees identified for the trial according to the Trial Monitoring Plan.
4. Expedited reporting of SUSARs/USADEs to the Competent Authority (MHRA in UK) and REC within required timelines – oversight, delegated to CRO
5. Notifying Investigators of SUSARs/USADEs that occur within the trial oversight, delegated to CRO.
6. Checking for (annually) and notifying PIs of updates to the Reference Safety Information for the trial.

Preparing standard tables and other relevant information for the DSUR in collaboration with the CI and ensuring timely submission to the MHRA and REC CRO

The appointed CRO will be responsible for project management of the trial (on behalf of the Sponsor). The CRO will be responsible for review of study documents to be submitted for ethics/regulatory approvals, tracking the progress of the study and timelines, SAE tracking and management, safety reporting to device sector, expedited safety reporting (SUSARs/USADEs), site initiation visit and site activation, study monitoring.

Trial Steering Committee (TSC):

In accordance with the Trial Terms of Reference for the TSC, periodically reviewing safety data and liaising with the DSMB regarding safety issues.

Data & Safety Monitoring Board (DSMB):

In accordance with the Trial Terms of Reference for the DSMB, periodically reviewing overall safety data to determine patterns and trends of events, or to identify safety issues, which would not be apparent on an individual case basis. The DSMB will be responsible for authorising dose escalation.

9.5 Notification of deaths

All deaths will be reported to the sponsor irrespective of whether the death is related to disease progression, the IMP, or an unrelated event. All deaths occurring during the study will be reported to the Sponsor by the investigator by emailing sponsor@uhs.nhs.uk. The CRO will be copied in the correspondence. For all deaths, available autopsy reports and relevant medical reports will be made available for reporting to the relevant authorities.

9.6 Pregnancy reporting

Pregnancy reporting will not be required for this study. Pregnancy is an exclusion criterion and will be confirmed at screening. As patients are hospitalised it is not expected that they will become pregnant during their participation in the treatment phase of this study.

9.7 Overdose

The definition of an overdose for this study is administration of one or more additional vials in relation to the dosing schedule displayed in section 8.6.

All reports of overdose with and without an adverse event must be reported by the investigator within 24 hours to the Sponsor (sponsor@uhs.nhs.uk). The CRO will be copied in the correspondence. If an SAE is associated with the overdose it will be fully described in the SAE report form by the investigator or delegate. An overdose will be recorded as a protocol deviation and recorded on the protocol deviation log.

9.8 Reporting urgent safety measures

If any urgent safety measures are taken the CI/Sponsor shall immediately and in any event no later than 3 days from the date the measures are taken, give written notice to the MHRA and the relevant REC of the measures taken and the circumstances giving rise to those measures.

9.9 The type and duration of the follow-up of participants after adverse reactions.

Patient's that experience an adverse reaction as a result of trial treatment will be followed up until day 28 or discharge from hospital. If additional follow up is required for safety reasons the investigator will discuss this with the participant and make appropriate arrangements. Adverse events will be recorded and reported for the duration of the patient's participation in the study. Any SUSAR will need to be

reported to the Sponsor irrespective of how long after IMP administration the reaction has occurred until resolved.

9.10 Development safety update reports

A Development Safety Update Report (DSUR) will be submitted by the Sponsor to the competent authority and ethics committee on the anniversary of the first approval date from the regulatory authority for the IMP.

10 STATISTICS AND DATA ANALYSIS

Feasibility

The adherence to per-protocol administration of Alveofact® will be summarized overall, and by dosing regimen:

- Screening, enrolment and consenting of patients
- Number and proportion of participants with initial dose successfully administered according to protocol
- Number and proportion of participants with collection of relevant intermediate physiological outcomes and safety outcomes
- Number and proportion of participants with administration of subsequent doses according to protocol criteria
- Number and proportion of participants with collection of efficacy outcome data
- Number and proportion of participants who completed the study per protocol

Safety

The incidence and 95% Confidence Intervals of SAEs, and Grade 3 or 4 AEs will be summarized by randomization group over the first 24 hours, and throughout the study. The number and percentage of participants with AEs will be summarized by Medical Dictionary for Regulatory Activities (MedDRA) system organ class (SOC) and preferred term (PT). Additional summaries will present the number and percentage of participants with adverse events by intensity and by relationship to surfactant treatment. Summary statistics will be generated for raw laboratory safety tests, as well as for any changes from baseline, as deemed clinically appropriate.

Efficacy

The goal of this study is to assess the preliminary efficacy and safety of surfactant administration, identify an efficacy signal quickly (if present) to consider initiation of a larger randomised controlled clinical trial, and assess the potential impact of total dose on preliminary efficacy.

The change in PaO_2/FiO_2 ratio relative to baseline at 24 hours after the last dose of surfactant and the change in Ventilation Index relative to baseline at 24 hours after the last dose of surfactant will be analysed separately. For each endpoint, a test for a total dose-response effect using a model-based multiple contrast test (Bretz et al, 2005) will be performed. Candidate dose-response models will include linear and Emax. The change in PaO_2/FiO_2 and change in Ventilation Index may be transformed for analysis (e.g., log transformed) to better satisfy normality assumptions of model residuals. The primary analysis will be done on patients with observed data at 24 hours ("completers"), though sensitivity analyses will also be performed if data are missing at 24 hours in an informative manner (e.g., due to death) where a non-parametric approach can accommodate missing data by assigning the worst rank.

The table below shows the power to show a significant dose response under varying assumed true dose response curves when using 12 matched controls.

CV	Relative change from baseline, on average					Power to show dose response
	Control	Cohort 1 (30 total vials)	Cohort 2 (50 total vials)	Cohort 3 (70 total vials)	Cohort 4 (90 total vials)	
20%	0%	0%	0%	0%	0%	0.05
	0%	10%	20%	30%	40%	0.89
	0%	25%	25%	25%	25%	0.82
	0%	0%	25%	25%	25%	0.75
	0%	0%	0%	30%	30%	0.72
	0%	0%	0%	0%	50%	0.63
25%	0%	0%	0%	0%	0%	0.05
	0%	10%	20%	30%	40%	0.77
	0%	25%	25%	25%	25%	0.66
	0%	0%	25%	25%	25%	0.57
	0%	0%	0%	30%	30%	0.54
	0%	0%	0%	0%	50%	0.49
Based on 2000 simulations for each row.						

When the true residual CV is 20%, there is reasonable power to detect a dose response for either endpoint when the true increase is at least 20-30% in two or more cohorts. There is limited power to detect a dose response when the effect is only in the highest cohort. Also, when the true residual CV is larger, the power reduces substantially. To reduce residual variability, the fitted model will also adjust for the baseline PaO_2/FiO_2 ratio or Ventilation Index as a proxy measure of baseline severity. Significant dose response model fits can be utilized to estimate doses that provide clinically meaningful results and aid in planning for future studies.

Efficacy data will be continuously reviewed after each cohort to assess whether results are compelling enough to suggest initiation of a larger randomised controlled clinical trial. Interim efficacy data will be analysed using a simple Bayesian adaptive approach. After each cohort completes dosing plus 24 hours of follow-up, the posterior distributions of the mean change in PaO_2/FiO_2 ratio and the mean change in Ventilation Index will be determined under an assumed normal distribution with a non-informative prior. The change in PaO_2/FiO_2 and change in Ventilation Index and may be transformed for analysis (e.g., log transformed) to better satisfy normality assumptions. Interim efficacy will be declared for the purposes of further trial planning if there is relatively high posterior probability (e.g., > 80%) that EITHER the true mean change in PaO_2/FiO_2 OR mean change in Ventilation Index at 24 hours after last administration of surfactant “high” (e.g., ≥20%), either for an individual cohort or for all treated patients combined.

The following table shows the probability of observing at least 80% posterior probability that the true improvement is ≥ 20% on average for each endpoint.

Relative change from baseline in each	Cumulative probability to observe at least 80% posterior probability that true change is ≥ 20% on average

group, on average	After 6 complete	After 9 complete	After 12 complete
0%	0.01	0.02	0.03
10%	0.06	0.14	0.19
20%	0.30	0.46	0.58
25%	0.45	0.64	0.75
30%	0.60	0.81	0.90
1000 simulations per row, assumes true CV = 20%			

When there is no true improvement, there is < 5% probability to declare efficacy at an interim time point. When the true improvement is 30% on average, there is 60% (81%) probability to declare efficacy at an interim time point after 6 (9) patients complete the study.

<u>Criteria for termination of the trial</u>

1. *Feasibility:*
    a. Failure to enrol patients
    b. Failure to administer drug (not necessarily full dose)
    c. Failure to collect primary outcome data
2. Safety:
    a. Any serious adverse event judged by the DSMB to justify trial termination
3. *Efficacy:*
    a. Clear evidence of harm resulting from the study intervention

11 DATA MANAGEMENT

11.1 Data collection tools and source document identification

Source Data

ICH E6 section 1.51, defines source data as "All information in original records and certified copies of original records or clinical findings, observations, or other activities in a clinical trial necessary for the reconstruction and evaluation of the trial. Source data are contained in source documents (original records or certified copies)."

Source Data Verification (SDV) will be undertaken as part of the monitoring activities for the trial. See Section 12 for further details.

Source Documents

Source documents include all original records of observations, results and activities necessary to reconstruct and evaluate the study. Source documents include but are not limited to laboratory reports, electrocardiogram tracings, hospital charts or pharmacy records, and any other records or reports of procedure performed during the study.

Case report forms

Patient data obtained during the study as a result of the study procedures, as well as relevant data from the medical history of the patient, findings as they occur, and other relevant data must be reported to the sponsor by the investigator. Data must be reported in electronic Case Report Forms (eCRF). Different accounts are available for investigator site staff (Data Coordinator and Investigator).

An eCRF will be designed in ALEA with assistance from the CI. The final eCRF will be approved by the sponsor. Site staff will be trained on completion and data entry requirements.

11.2 Data management

The data will be managed with support from the Clinical Informatics Research Unit using ALEA eCRF® (FormsVision BV, Netherlands). This electronic CRF (eCRF) service for data collection and management provides comprehensive, user friendly forms service which can be used with a standard browser running on any computer connected to the internet. The system has been validated and has been certified by registered auditors to be in compliance with regulation, such as the FDA's CFR 21 Part 11 and ICH GCP.

Anonymous data will be available for request from three months after publication of the article, to researchers who provide a completed Data Sharing request form that describes a methodologically sound proposal, for the purpose of the approved proposal and if appropriate, signed a Data Sharing Agreement. Proposals will be reviewed by the study team. Data will be shared once all parties have signed relevant data sharing documentation, covering the study team conditions for sharing and if required, an additional Data Sharing Agreement from Sponsor. Proposals should be directed to the chief investigator.

A data management plan will be in place once this protocol has been finalised.

At the end of the study and with sponsor approval, the database will be locked. The statistician has access to the database who will undertake the data analysis following a statistical analysis plan.

All data Management activities will be undertaken in accordance with CIRU SOPs that will be adhered to by all study staff.

Primary data will be entered into the eCRF by delegated study team members. Primary sources of laboratory data will be from hospital computerised systems and recorded in the database accordingly.

Data queries can be raised from within ALEA and addressed by the site once the query has been raised.

Source documents are where data are first recorded, and from which participants' Case Report Form (CRF) data are obtained.

11.3 Data handling and record keeping

Data will be collected and retained in accordance with local laws and regulations, for example the General Data Protection Regulation (2018) in the UK. Apart from questionnaires, the investigator at each site is responsible for ensuring the accuracy, completeness, and timeliness of the data.

Study documents will be retained in a secure location during and after the study has finished. During the study, all data will be reported in pseudonymised form and will be identified by the assigned participant number. Individual sites will only have access on ALEA to data collected via their specific site, including that which links a participant to their assigned participant number. The administrative centre will have access via ALEA to all data including the participant identifiable details, from all international sites.

Only the Site Investigator and authorised personnel should enter or change data in the electronic Case Report Forms (eCRFs) in ALEA. An audit trail will be incorporated into the eCRFs whereby any changes to the data originally entered will be documented. A table of all changes including the original value, new value, field, relevant visit details, who made the changes and why the changes were made, will be stored in a table in the study database.

Quality of the data entered into the eCRF data fields will be controlled by limited data entry, drop down options and predefined data formats. Range checks for chosen fields will automatically appear where data points are outside of a pre-specified range. Verification and explanation for the data point will be required and will subsequently appear in the query log for the Data Manager or Study Manager to check.

11.4 Access to Data

Direct access will be granted to authorised representatives from the Sponsor, host institution and the regulatory authorities to permit trial-related monitoring, audits and inspections- in line with participant consent.

11.5 Archiving

Archiving will be authorised by the Sponsor following submission of the end of study report.

Location and duration of record retention for:

- Essential documents: Patient case notes will be stored and maintained according to standard rules and procedures. Pathology results are stored and maintained according to standard procedures.
- The trial database: The trial database will be held by the vendor for a minimum period of 5 years from the completion of the study. A copy will be sent to the PI once the database has been decommissioned following the relevant CIRU SOP Destruction of essential documents will require authorisation from the Sponsor.

12 MONITORING, AUDIT & INSPECTION

The monitoring activities for the trial will be undertaken by the CRO. A trial specific monitor (CRA) will be assigned to the trial and a monitoring plan will be established and agreed by the sponsor. A risk-based approach to monitoring has been adopted for the trial. Monitoring visits will be required prior to a dose escalation decision being made. 100% source data verification (SDV) will take place for key study parameters and data used as part of the dose escalation decision making process (Pa02/Fi02 ratio and Ventilation index at 48 hours) as outlined in the MP.

All monitoring reports will be sent to the sponsor for review following each monitoring activity. Monitoring reports are subject to review by the DSMB and TSC.

Auditing activities will be the responsibility of the sponsor.

13 ETHICAL AND REGULATORY CONSIDERATIONS

13.1 Research Ethics Committee (REC) review & reports

The sponsor will ensure that the trial protocol, patient information sheet, consent form, GP letter and submitted supporting documents have been approved by the appropriate regulatory body (MHRA in UK), Health Research Authority (HRA), main research ethics committee (REC) and that local permission has been obtained prior to any participant recruitment.

All substantial amendments (as determined by the sponsor) will not be implemented until HRA/REC and/or the MHRA have provided the relevant authorisations. The NHS R&D departments will also be informed of any substantial amendments. Relevant approvals must be obtained before any substantial amendment may be implemented at sites.

All correspondence with the HRA, REC and the MHRA will be retained in the Trial Master File and the Investigator Site File (maintained by the site).

An annual progress report (APR) will be submitted to the REC (by the site) within 30 days of the anniversary date on which the favourable opinion was given, and annually until the trial is declared ended.

Within 90 days after the end of the trial (as defined in section 5.8), the CI/Sponsor will ensure that the HRA/ main REC and the MHRA are notified that the trial has finished. If the trial is terminated prematurely, those reports will be made within 15 days after the end of the trial.

The CI will supply the Sponsor with a summary report of the clinical trial, which will then be submitted to the MHRA and main REC within 1 year after the end of the trial.

All results will be published on a publicly accessible database (EUDRACT) in accordance with UK regulatory requirements.

13.2 Peer review

Peer review and scientific review has been undertaken by Professor Stephen Brett (Imperial) and Dr Ravi Gill (Southampton). Both peer reviews approved the study and gave an overall rating of 'excellent' to its scienfitic value.

13.3 Public and Patient Involvement

Due to the understandable urgency of the research application and research participants' grave illness, Public and Patient Involvement (PPI) was not feasible throughout. The focus was therefore on PPI for key information for patients/their legal representatives in order to make giving consent more understandable for people at such a very distressing time.

PPI was undertaken by someone with a relevant background in it who was involved in drafting Information Sheets. She contacted friends/acquaintances, explaining that a fast turn-around (24 hours) was needed and remuneration was not possible. Everyone approached agreed to help. Representativeness is complex and contested but opinions were obtained from seven people:

1. Someone who spent a lengthy period of time in ICU being ventilated with ARDS a few years ago and his wife and daughter. Another daughter has recently recovered from COVID-19
2. Someone with Idiopathic Pulmonary Fibrosis whose wife also has respiratory problems
3. A retired nurse and midwife with a long-standing interest in surfactant from her practice and personal experience who has recently recovered from COVID-19
4. An Imam at a local Mosque

Everyone was e-mailed:

1. A letter of explanation giving spaces to answer questions/make comments
2. Legal representative Information Sheet
3. Legal representative Consent Form
4. Patient Information Sheet

Most replies were e-mailed. One person was unable to print the form so collected it and it was later collected from her (both during permitted daily walks!) People's responses varied in length from no suggested amendments to specific changes and comments on the overall style. Responses were collated and all incorporated in document revision. All comments are valuable and, if one person experiences something as unclear/confusing, so might the person be receiving the actual document. The Personal Legal representative and Consent forms were amended to take account of the

responses from the PPI panel. People were sent revisions with an apology that there was no time for further comments.

It may be feasible to incorporate PPI at a later stage in dissemination of findings.

.

13.4 Regulatory Compliance

The trial will not commence until a Clinical Trial Authorisation (CTA) is obtained from the MHRA, a favourable opinion obtained from the REC and HRA approval is obtained. Local capacity and capability will be obtained before any participant activity is undertaken.

For any amendment that will potentially affect a site's capacity and capability, the Principal Investigator(s) or designee will confirm with that site's R&D department that capacity and capability is ongoing.

The protocol and trial conduct will comply with the Medicines for Human Use (Clinical Trials) Regulations 2004/SI 1031 and any relevant amendments.

13.5 Protocol compliance

The Investigator agrees to comply with the requirements of the Protocol and Good Clinical Practice. Prospective, planned deviations or waivers to the protocol are not allowed under the UK regulations on Clinical Trials and must not be used e.g. it is not acceptable to enrol a subject if they do not meet the eligibility criteria or restrictions specified in the trial protocol.

Accidental protocol deviations can happen at any time. They must be adequately documented on the relevant forms and reported to the Chief Investigator and Sponsor immediately.

Deviations from the protocol, which are found to frequently recur, are not acceptable and will require immediate action by the sponsor. Frequent non-compliances could potentially be classified as a serious breach (see section 13.6).

13.6 Notification of Serious Breaches to GCP and/or the protocol

A "serious breach" is a breach which is likely to effect to a significant degree:

(a) the safety or physical or mental integrity of the subjects of the trial; or

(b) the scientific value of the trial

The sponsor using sponsor@uhs.nhs.uk will be notified immediately of any case where the above definition applies during the trial conduct phase and will notify the MHRA GCP Inspectorate using the following email address:

GCP.SeriousBreaches@mhra.gov.uk

and REC in writing of any serious breach of:

(a) the conditions and principles of GCP in connection with that trial; or

(b) the protocol relating to that trial, as amended from time to time

(c) within 7 days of becoming aware of that breach.

The MHRA template form for notifications of serious breaches to the MHRA will be used to ensure all appropriate information is submitted to the GCP Inspectorate.

13.7 Data protection and patient confidentiality

All investigators and trial site staff will comply with the requirements of the Data Protection Regulation with regards to the collection, storage, processing and disclosure of personal information and will uphold the Regulation's core principles.

The Case Report Forms (CRFs) will not contain subject names or other personal identifiable data. The subject's initials, date of birth and trial identification number, will be used for identification.

No subject identifiable data will be transferred to the sponsor or data management vendor.

The sites will hold a master subject list (with identifiable information) that will remain in the site file and on password protected computer systems only.

13.8 Financial and other competing interests for the chief investigator, PIs at each site and committee members for the overall trial management

The investigators for the trial have no competing interests relevant to the IMP, device or overall trial. No committee members have relevant competing interests to be declared at the time of protocol writing.

13.9 Indemnity

The NHS Negligence Scheme will apply for this study.

13.10 Amendments

Under the Medicines for Human Use (Clinical Trials) Regulations 2004, the sponsor may make a non-substantial amendment at any time during a trial. If the sponsor wishes to make a substantial amendment to the CTA or the documents that supported the original application for the CTA, the sponsor must submit a valid notice of amendment to the licensing authority (MHRA) for consideration. If the sponsor wishes to make a substantial amendment to the REC application or the supporting documents, the sponsor must submit a valid notice of amendment to the REC for consideration. The MHRA and/or the REC will provide a response regarding the amendment within 35 days of receipt of the notice. It is the sponsor's responsibility to decide whether an amendment is substantial or non-substantial for the purposes of submission to the MHRA and/or REC.

Amendments need to be notified to NHS R&D departments of participating sites to assess whether the amendment affects the capacity and capability for that site.

All amendments must be notified to the HRA and CRN for categorisation. Implementation will be based upon the amendment category assigned.

Version control will be maintained for any amendments made. Substantial Amendments (SAs) will be identified by a whole number change (i.e. v1.0, v2.0) and non-substantial amendments (NSAs) identified by a decimal change (i.e. v1.1, v1.2). All amendments will be logged at each site and a central copy maintained in the TMF.

Guidance on the categorisation of amendments can be found on the HRA website http://www.hra.nhs.uk/resources/after-you-apply/amendments/

13.11 Post trial care

No interventions or benefit related to the study will be provided to participants once the study has completed. Patients will follow routine clinical care once their participation in the study has ended.

14 DISSEMINIATION POLICY

14.1 Dissemination policy

On completion of the study a final check on the data will be performed and any missing data collected or otherwise accounted for. Data will be tabulated and analysed as described in the statistical plan. A final study report will be prepared by the CI for submission to the REC, MHRA, Sponsor and R&D. The time period from the completion of follow-up of the last trial subject and submission of the report will follow regulatory guidelines. All publications relating to the study will follow Consort Guidelines.

It is anticipated that the study will be prepared for submission to meetings and for publication. Any publication must have the approval of the CI/PI. There will be no time limit for the production of any publication.

Participants in the study will be informed of the final results if appropriate to do so. Participants will be informed following the preparation of the final report and may be provided with a lay person's summary of the conclusions.

14.2 Authorship eligibility guidelines and any intended use of professional writers

The list of authors will consist of those individuals who, at the discretion of the CI, have contributed to the creation of the study itself, to the recruitment and care of subjects, to the collection and analysis of the data, to the preparation of any reports, abstracts and commentary and final publication of the study.

15 REFERENCES

Anzueto A, Baughman RP, Guntupalli KK, Weg JG, Wiedemann HP, Raventos AA, Lemaire F, Long W, Zaccardelli DS, Pattishall EN: Aerosolized surfactant in adults with sepsis-induced acute respiratory distress syndrome. Exosurf Acute Respiratory Distress Syndrome Sepsis Study Group. N Engl J Med 1996, 334:1417-21.

Surfactant replacement therapy for severe neonatal respiratory distress syndrome: an international randomised clinical trial. Collaborative European Multicenter Study Group. Pediatrics 1988, 82:683-91.

Dushianthan A, Goss V, Cusack R, Grocott MP, Postle AD: Altered molecular specificity of surfactant phosphatidycholine synthesis in patients with acute respiratory distress syndrome. Respir Res 2014, 15:128.

Goss V, Hunt AN, Postle AD: Regulation of lung surfactant phospholipid synthesis and metabolism. Biochim Biophys Acta 2013, 1831:448-58.

Gunther A, Schmidt R, Harodt J, Schmehl T, Walmrath D, Ruppert C, Grimminger F, Seeger W: Bronchoscopic administration of bovine natural surfactant in ARDS and septic shock: impact on biophysical and biochemical surfactant properties. Eur Respir J 2002, 19:797-804.

Hoffmann M, Kleine-Weber H, Schroeder S, Kruger N, Herrler T, Erichsen S, Schiergens TS, Herrler G, Wu NH, Nitsche A, Muller MA, Drosten C, Pohlmann S: SARS-CoV-2 Cell Entry Depends on ACE2 and TMPRSS2 and Is Blocked by a Clinically Proven Protease Inhibitor. Cell 2020,181, 1:10.

Moller JC, Schaible T, Roll C, Schiffmann JH, Bindl L, Schrod L, Reiss I, Kohl M, Demirakca S, Hentschel R, Paul T, Vierzig A, Groneck P, von SH, Schumacher H, Gortner L: Treatment with bovine surfactant in severe acute respiratory distress syndrome in children: a randomized multicenter study. Intensive Care Med 2003, 29:437-46.

Postle AD, Mander A, Reid KB, Wang JY, Wright SM, Moustaki M, Warner JO: Deficient hydrophilic lung surfactant proteins A and D with normal surfactant phospholipid molecular species in cystic fibrosis. Am J Respir Cell Mol Biol 1999, 20:90-8.

Qi F, Qian S, Zhang S, Zhang Z: Single cell RNA sequencing of 13 human tissues identify cell types and receptors of human coronaviruses. Biochem Biophys Res Commun 2020 https://doi.org/10.1016/j.bbrc.2020.03.044.

Rebello CM, Jobe AH, Eisele JW, Ikegami M: Alveolar and tissue surfactant pool sizes in humans. Am J Respir Crit Care Med 1996, 154:625-8.

Rodriguez-Capote K, Manzanares D, Haines T, Possmayer F: Reactive oxygen species inactivation of surfactant involves structural and functional alterations to surfactant proteins SP-B and SP-C. Biophys J 2006, 90:2808-21.

Schmidt R, Markart P, Ruppert C, Wygrecka M, Kuchenbuch T, Walmrath D, Seeger W, Guenther A: Time-dependent changes in pulmonary surfactant function and composition in acute respiratory distress syndrome due to pneumonia or aspiration. Respir Res 2007, 8:55.

Schwarz KB: Oxidative stress during viral infection: a review. Free Radic Biol Med 1996, 21:641-9.

Shi H, Han X, Jiang N, Cao Y, Alwalid O, Gu J, Fan Y, Zheng C: Radiological findings from 81 patients with COVID-19 pneumonia in Wuhan, China: a descriptive study. Lancet Infect Dis 2020, 20:425-34

Walmrath DA, Gunther HA, Ghofrani R, Schermuly T, Schneider F,Grimminger, W. Seeger: Bronchoscopic surfactant administration in patients with severe adult respiratory distress syndrome and sepsis. Am J Respir Crit Care Med 1996, 154: 57-62.

Walmrath D, Grimminger F, Pappert D, Knothe C, Obertacke U, Benzing A, Gunther A, Schmehle T, Leuchte H, Seeger W. Bronchoscopic administration of bovine natural surfactant in ARDS and septic shock: Impact on gas exchange and haemodynamics. Eur Respir 2002 J 19:805–810.

Westhoff M, L Freitag: Surfactant--treatment of complete lobar atelectasis after exacerbation of bronchial asthma by infection. Pneumologie 2001, 55:130-134.

WHO master protocol 2020 https://www.who.int/docs/default-source/coronaviruse/situation-reports/20200301-sitrep-41-covid-19.pdf?sfvrsn=6768306d_2

16. APPENDICES

16.1 Appendix 1

Schedule of Procedures Screening – Day 13

Procedures		Baseline / Treatment Phase				Follow Up											
	Screening	D0	8 Hours	16 Hours	24 Hours	(48 hrs)	(72 hrs)	D4	D5	D6	D7	D8	D9	D10	D11	D12	D13
Informed consent	X																
Inclusion / Exclusion	X																
Demographics	X																
Medical history	X																
Urine Pregnancy Test	X																
Vital signs & Ventilation Parameters[1]	X	X	X	X	X	X	X	X	X	X	X	X	X	X	X	X	X
Concomitant medications	X	X	X	X	X	X	X	X	X	X	X	X	X	X	X	X	X
Diagnostic PCR for SARS-CoV-2 in TA							X		X					X			
Tracheal aspirate collection[2]		X	X	X	X	X	X										

[1] Measurements will be taken as part of routine clinical care. Results will be entered into the eCRF for the purposes of the study. Measurements will be recorded in between the administration of each vial of surfactant and hourly between each dose and until 30 hours. Measurements will then be taken 6 hourly until 48 hours and then daily.

[2] Baseline samples of lung TA samples will be collected up to 30 minutes before administration of the first dose of surfactant (t=0h). Samples will then be collected up to 15 minutes before the second (t=8h) and third (t=24h) doses of surfactant, at 16 hours and at 48 and 72 hours thereafter if the patient is still intubated.

Procedures	Baseline / Treatment Phase		Follow Up														
	Screening	D0	8 Hours	16 Hours	24 Hours	(48 hrs)	(72 hrs)	D4	D5	D6	D7	D8	D9	D10	D11	D12	D13
Blood sample collection		X	X	X	X	X	X										
Randomisation		X															
Arterial Blood Gasses sampling		Samples will be taken at 0, 1, 2, 3, 6, 8, 9, 10, 11, 14, 18, 24, 25, 26, 27, 30, 36 hours															
Nebuliser Administration[3]		X	X		X												
Surfactant Administration[4]		X	X		X												
Infusion of methyl-D9-choline chloride		X[5]															
Clinical Monitoring (as per SoC)	X	Patients will be monitored hourly up to 32 hours post 1st dose				X[6]	X	X	X	X	X	X	X	X	X	X	X
Chest Ultrasound[7]	X	X	X	X	X	X	X	X	X	X	X	X	X	X	X	X	X
Adverse event assessments		X	X	X	X	X	X	X	X	X	X	X	X	X	X	X	X

---

[3] Patients randomised to the treatment arm only
[4] Patients randomised to the treatment arm only
[5] Infusion of methyl-D_9-choline chloride will be initiated during the stabilization period between intubation and administration of the first dose of surfactant
[6] 4 hourly until 48 hours post first dose
[7] A chest ultrasound will be performed before and after each dose of surfactant. On all other occasions the chest ultrasound is performed daily.

Day 14-28

Procedures	Follow Up														
	D14	D15	D16	D17	D18	D19	D20	D21	D22	D23	D24	D25	D26	D27	D28
Vital signs & Ventilation Parameters	X	X	X	X	X	X	X	X	X	X	X	X	X	X	X
Concomitant medications	X	X	X	X	X	X	X	X	X	X	X	X	X	X	X
Diagnostic PCR for SARS-CoV-2 in TA		X													X
Clinical Monitoring (as per SoC)	X	X	X	X	X	X	X	X	X	X	X	X	X	X	X
Chest Ultrasound	X	X	X	X	X	X	X	X	X	X	X	X	X	X	X
Adverse event assessments	X	X	X	X	X	X	X	X	X	X	X	X	X	X	X

16.2 Appendix 2 – Amendment History

Amendment No.	Protocol version no.	Date issued	Author(s) of changes	Details of changes made
1	2.0	05/06/2020	Josune Olza Meneses<br><br>Tony Postle	Page 11:Study start date changed from May 2020 to June 2020.<br><br>Page 20: Secondary endpoint days changed from 29 to 28<br><br>Page 21: Added - Reduction of inflammatory indices such as cellular and cytokine inflammatory markers and of oxidative stress markers in blood samples to Exploratory Endpoints.<br><br>Page 21: Changed 3.3.3 5. from Reduction of inflammatory indices such as cellular and cytokine inflammatory markers in TA and in blood samples and of oxidative stress makers in blood samples to Reduction in inflammatory indices such as cellular and cytokine inflammatory markers in TA samples<br><br>Page 24: Removed blood samples from point 5<br><br>Page 24: Added Oxaditive Stress Markers and 16 hours to point 7<br><br>Page 30: Added information of how subject identifiers will be created<br><br>Page 31: Clarification of Demographic Information identifiers<br><br>Page 34: Baseline samples: Lung TA samples will now be collected within 15/30 minutes rather than immediately<br><br>Page 35: BAL have been changed to TA samples.<br><br>Page 41: Definition of SAE has been removed<br><br>Page 45: Addition of device or device deficiency rather than just device in the determining of relationship to IMP<br><br>Page 46: Now only one table for assessment of causality rather than separate table of for IMP and device<br><br>Page 47: Addition of the use of the Device Deficiency Form which must be

				used to report any serious events involving the device<br>Page 51: The SAEs must now be assessed for expectedness against the IB<br>Page 51: CRO oversight removed from the preparation of DSURs<br>Pages 65-68: Changes made to timings on table of procedures and Traceal Aspirate and PCR collection changed to just Traceal Aspirate collection.<br>All references to Consultee have been changed to either Legal Representative or Professional Representative.<br>All references to 'Directions for Use' have been changed to 'Instructions for Use'
